# Supplementary material for: Allosteric regulation and crystallographic fragment screening of SARS-CoV-2 NSP15 endoribonuclease
Source: Nucleic Acids Res. 2023 Apr 28;51(10):5255–70. doi: 10.1093/nar/gkad314 (PMC10250223; doi:10.1093/nar/gkad314)
Supplement: gkad314_Supplemental_Files [file gkad314_supplemental_files.zip › Supplementary datasets.zip!/188/Refine_9/01-BUSTER/Cycle-4/LIST.html]

Refining

**BUSTER 2.X**: A suite of programs for macromolecular structure refinement by maximum-likelihood and Bayesian methods  
Copyright © 1992-2020 Global Phasing Ltd.  
All rights reserved.  
Authors: G. Bricogne, E. Blanc, M. Brandl, C. Flensburg, P. Keller, W. Paciorek, P. Roversi, O. Smart, C. Vonrhein, T. Womack  

Please reference use of the software as:

- BUSTER-TNT 2.X, Global Phasing Ltd, Sheraton House, Cambridge CB3 0AX, UK

Version: 2.10.3 for linux64-ifort made on May 20 2020 at 01:14:34  
Licenced to: Diamond Light Source, Scientific Software  

```
---


### Parsing of the BUSTER cardfile

TITLE : autoBUSTER refinement

   Cell information

   Symmetry information

 Using CCP4 X-ray form factors.

   Missing structure chemical composition

 Using ATOM block for chemical composition.


   Shell information

### Parsing of the TNT cardfile


---


### Reading observations file

 Number of reflexions in input:  47008

      Number of accepted reflexions:  40456

      Number of rejected reflexions:      0

   Reflexions in test set:            2022

   Reflexions in working set:        38434


---


### 1. Generating initial model

 2.10 * Shannon sampling rate. NX NY NZ:   288  288  192


  Effective Shannon rates are:    2.44    2.44    2.23


   Allocated BUFFER of size:     61.172 Mb.

   Initial Geometry Information


 No missing atoms declared


   Summary of model volumes

 No missing atoms declared: no prior computed


 Computing bulk solvent model scattering


 NCSMASK PDB radius for solvent mask:     2.15 Angstrom

 running PDBSET (setting cell and spacegroup P1) ... done
 running NCSMASK [1] (creating mask, SMOOTH 1.14073) ... done
 running MAPDUMP (to check if reduction to unit cell is needed) ... done
 running MAPMASK (converting mask to map) ... done

   Generation of babslv by theta-filter

   Electron density in frg mask:  0.420 Å-1

 FOBS  put on rough absolute scale by Wilson plot :
           K        =  0.69668E+02
           B        =    -10.511

 

### Cycle 0001: MaxLik scaling and refinement


   MaxLik Scaling Cycle 0001

   Wilson plot

   Statistics in resolution bins just after scaling
```

|  |  |
| --- | --- |
|  | - Rxpct factors plot- Rxpct factors table- <Log-Lik Gain>- Ln Sigma A- Correlation Coefficients |

```
   <Log-Likelihood> of null-hypothesis

Structure Factor Amplitudes


   Average Figure of Merit

 Switching off refinement of overall temperature factor


 Added B =     0.1095 Å2 to all atoms in fragment


 Added B =     0.1095 Å2 to B_SOLV

```
gelly refinement module
 Authors (2004-2009) O. S. Smart, C. Flensburg, W. Paciorek, C. Vonrhein, 
                     M. Brandl, T. O. Womack and G. Bricogne
         (2010-2011) O. S. Smart, C. Flensburg, W. Paciorek, C. Vonrhein, 
                     T. O. Womack and G. Bricogne
         (2012-2014) O. S. Smart, C. Flensburg, W. Paciorek, C. Vonrhein, 
                     and G. Bricogne
         (2015-2020) G. Bricogne, C. Flensburg, W. Paciorek, C. Vonrhein
 Copyright © 2004-2020 Global Phasing Ltd. 
 All rights reserved.
 gelly module compilation date May 20 2020 01:11:20
 
 gelly uses routines from TNT geometry. References: 
     Tronrud, D. E., Ten Eyck, L. F., Matthews, B. W. (1987)
     "An Efficient General-Purpose Least-Squares Refinement Program 
     for Macromolecular Structures". Acta Crystallogr A, 43:489-501.
     Tronrud, D. E. (1997). "The TNT Refinement Package", 
     in Macromolecular Crystallography, Part B, Eds Charlie Carter 
     and Robert Sweet, Methods in Enzymology, 277:306-319.
 wildcard matching function by Julian Robichaux http://www.nsftools.com

 No -random_seed flag found. Random number stream initialised using:
2147483562
2147483398

 Control flags set to:  -autoncs -hydrogens_input_occ2zero \
                     -hydrogens_output_occ2heavy -screen \
                     100 -screen_sigma \
                     3.0 -keeppdb \
                     1000000 -taomonitor_verbose \
                     2 
 

 ############### Starting setup via TNT routines

 Setting up tnt routines including license check:

                   TNT Stereochemistry Program
 Version: 5.22.3.0
 Licence for Diamond Light Source, Scientific Software is valid
 
 Using TNT geometry routines to load the tnt control file: gelly.tnt
 Using TNT routines to load initial coordinates from file fragment.cor
 Expecting 6519 atoms

 Found     2194 "NOTE BUSTER_RESIDUE_ATOM_DATA" cards
 Have copied information for    6519 atoms from TNT to gelly's store 
 
 The TNT WEIGHT cards used by this run:
 -----WEIGHT       BOND     2.0
 -----WEIGHT       ANGLE    2.0
 -----WEIGHT       IMPROPER 0.0
 -----WEIGHT       TORSION  2.0
 -----WEIGHT       PSEUDO   0.0
 -----WEIGHT       TRIGONAL 2.0
 -----WEIGHT       PLANE    5.0
 -----WEIGHT       CONTACT  5.0
 -----WEIGHT       BCORREL  10.0
 -----WEIGHT       CHIRAL   5.0
 -----WEIGHT       NCS      50.0
 -----WEIGHT       IDEAL    4.0
 -----WEIGHT       RFACTOR  9.48
 
 Dictionary versions picked up from NOTE BUSTER_RESTRAINT_DICT cards as: 
 ------ protgeo_eh99.dat (V1.6.4.17) 20191001  STANDARD AMINO ACID DICTIONARY. BONDS AND ANGLES FROM ENGH AND HUBER EH99. OTHER VALUES BASED ON PREVIOUS TNT OR TAKEN FROM CCP4. INCLUDES HYDROGEN ATOMS.
 ------ exoticaa.dat (V1.3.4.15) 20200327  COLLECTION OF NON-STANDARD AMINO ACIDS, MAINLY EH91 WITHOUT IDEAL DISTANCE INFO
 ------ nuclgeo.dat (V1.13.4.8) 20190909
 ------ bcorrel.dat (V1.15.10.2) 20190616
 ------ contact.dat (V1.15.12.10) 20191121
 ------ idealdist_contact.dat (V1.3.4.12) 20191001  IDEAL-DISTANCE CONTACT TERM DATA AS USED IN PROLSQ. VALUES USED HERE ARE BASED ON DATA HARVESTING OF PDB AS OF 20190109.
 ------ restraints for LIG (Unknown) from cif dictionary Z1262327505.cif
 ------ restraints for CIT (CITRIC ACID) from cif dictionary CIT.cif; generated by GRADE_PDB_LIGAND 1.2.12 (October 4 2016) using MOGUL 1.7.2(RC1), CSD as538be, with quantum mechanics RM1 ((DatabaseID PDB-CIT))
 ------ assume.dat (V1.9.4.2) 20180705
 
 Have picked up spacegroup name for pdb output as 'P 63      '
 Setting up Chain/Residue arrays
 
 Have command line specification -autoncs*
     Have found       1 NCS relations
     Pairwise matching residue number and type between chains: 
                    A        B        S        W    
          A         .      100%       -        -   
          B                  .        -        -   
          S                           .        -   
          W                                    .   
       Table key--                                                       
          all   : two chains have 100% match and same number of residues 
         multi  : ditto and they contribute to a multi chain ncs cluster 
          ***%  : two chains have >80% matching residue number/type      
           -    : chains appear to be unrelated                          
      chain A     have identified ncs to     1 other chains. So will weight each autoncs by (       6.666/#)=      6.6660
      chain B     have identified ncs to     1 other chains. So will weight each autoncs by (       6.666/#)=      6.6660
      chain S     have identified ncs to     0 other chains. 
      chain W     have identified ncs to     0 other chains. 
     For .Gelly cards produced see below - after pruning
 
 Setting up BUSTER_SET groups
 Have found    89 BUSTER_SET cards
 Now silently applying automatic/dictionary set definitions. For a verbose listing use '-verbose_set' flag.
 Applying user defined set definitions:
 =====NOTE BUSTER_SET ncsautoXcld = Water --> set 'ncsautoXcld' now has   966 atoms 
 =====NOTE BUSTER_SET ncsautoABset = Chain_A \ ncsautoXcld --> set 'ncsautoABset' now has  2783 atoms 
 Have defined    49 sets: 
      set:                 'All' has   6519 atoms in   1667 residues - automatically defined
      set:               'Empty' has      0 atoms in      0 residues - automatically defined
      set:            'ExoticAA' has      0 atoms in      0 residues - defined by dictionary file
      set:           'ExoticNUC' has      0 atoms in      0 residues - defined by dictionary file
      set:              'NotHET' has   5514 atoms in    698 residues - defined by dictionary file
      set:             'Chain_A' has   2783 atoms in    351 residues - automatically defined
      set:             'Chain_B' has   2770 atoms in    350 residues - automatically defined
      set:             'Chain_S' has    567 atoms in    567 residues - automatically defined
      set:             'Chain_W' has    399 atoms in    399 residues - automatically defined
      set:               'Water' has    966 atoms in    966 residues - defined by dictionary file
      set:          'StdProtein' has   5514 atoms in    698 residues - defined by dictionary file
      set:             'Protein' has   5514 atoms in    698 residues - defined by dictionary file
      set:                'Back' has   2794 atoms in    698 residues - defined by dictionary file
      set:                'Side' has   2720 atoms in    650 residues - defined by dictionary file
      set:        'RnaCanonical' has      0 atoms in      0 residues - defined by dictionary file
      set:        'DnaCanonical' has      0 atoms in      0 residues - defined by dictionary file
      set:     'RnaDnaCanonical' has      0 atoms in      0 residues - defined by dictionary file
      set:     'DnaRnaCanonical' has      0 atoms in      0 residues - defined by dictionary file
      set:         'RnaExtended' has      0 atoms in      0 residues - defined by dictionary file
      set:         'DnaExtended' has      0 atoms in      0 residues - defined by dictionary file
      set:      'RnaDnaExtended' has      0 atoms in      0 residues - defined by dictionary file
      set:      'DnaRnaExtended' has      0 atoms in      0 residues - defined by dictionary file
      set:                 'Dna' has      0 atoms in      0 residues - defined by dictionary file
      set:                 'Rna' has      0 atoms in      0 residues - defined by dictionary file
      set:              'RnaDna' has      0 atoms in      0 residues - defined by dictionary file
      set:              'DnaRna' has      0 atoms in      0 residues - defined by dictionary file
      set:             'Nucleic' has      0 atoms in      0 residues - defined by dictionary file
      set:             'Polymer' has   5514 atoms in    698 residues - defined by dictionary file
      set: 'BufferWeichenberger' has     26 atoms in      2 residues - defined by dictionary file
      set:    'BufferAdditional' has      0 atoms in      0 residues - defined by dictionary file
      set:              'Buffer' has     26 atoms in      2 residues - defined by dictionary file
      set:         'MetalAlkali' has      0 atoms in      0 residues - defined by dictionary file
      set:  'MetalAlkalineEarth' has      0 atoms in      0 residues - defined by dictionary file
      set:     'MetalTransition' has      0 atoms in      0 residues - defined by dictionary file
      set: 'MetalPostTransition' has      0 atoms in      0 residues - defined by dictionary file
      set:    'MetalLanthanides' has      0 atoms in      0 residues - defined by dictionary file
      set:               'Metal' has      0 atoms in      0 residues - defined by dictionary file
      set:         'SugarAlphaD' has      0 atoms in      0 residues - defined by dictionary file
      set:         'SugarAlphaL' has      0 atoms in      0 residues - defined by dictionary file
      set:               'Sugar' has      0 atoms in      0 residues - defined by dictionary file
      set:               'Other' has     39 atoms in      3 residues - defined by dictionary file
      set:            'Hydrogen' has      0 atoms in      0 residues - defined by dictionary file
      set:         'nonHydrogen' has   6519 atoms in   1667 residues - defined by dictionary file
      set:             'OccZero' has      0 atoms in      0 residues - defined by dictionary file
      set:          'OccNonZero' has   6519 atoms in   1667 residues - defined by dictionary file
      set:             'OccFull' has   6474 atoms in   1664 residues - defined by dictionary file
      set:          'OccNonFull' has     45 atoms in      3 residues - defined by dictionary file
      set:         'ncsautoXcld' has    966 atoms in    966 residues - user specified
      set:        'ncsautoABset' has   2783 atoms in    351 residues - user specified
 No command line arguments '-occ_allhet' or '-occ_restyp' so will not setup automated occcupancy refinement
 
 Interpreting CONSTANT and FREE cards: 
 =====CONSTANT     OCC
 Have found and interpreted      1 CONSTANT cards in the TNT input. 
 Before any COMBINE - Number of atoms with fixed XYZ =     0
 Before any COMBINE - Number of atoms with fixed B   =     0
 Before any COMBINE - Number of atoms with fixed OCC =  6519
 Before any COMBINE - Number of atoms with fixed BIJ =   966
                            cf total number of atoms =  6519
 
 There are no COMBINE cards to be found in the TNT card input 
 
 There are no NOTE BUSTER_COMBINE_RES cards to be found in the TNT card input 

 ############### Working out full connectivity list


 ############### Working out equivalent atoms


 Residue with most atoms given below:
        A|58:N(TRP)         
        A|58:CA(TRP)        
        A|58:CB(TRP)        
        A|58:CG(TRP)        
        A|58:CD1(TRP)       
        A|58:NE1(TRP)       
        A|58:CE2(TRP)       
        A|58:CD2(TRP)       
        A|58:CE3(TRP)       
        A|58:CZ3(TRP)       
        A|58:CH2(TRP)       
        A|58:CZ2(TRP)       
        A|58:C(TRP)         
        A|58:O(TRP)         


 Have no '-equiv_check_plus' argument - so will take atom type into
 account when checking for equivalence of otherwise identical atoms.
 

 ############### Looking at dihedrals


 ############### Looking at chirals

 NOTE BUSTER_CHIRAL_ANGLECUT card found - read value=    -31.00 degs from it
 NOTE BUSTER_CHIRAL_SIGMA card found - read value=      1.70 degs from it
 RANGE allowed for B variables in refinement:   from      3.00000 to    300.00000

 ############### Preparing for PDB output


 ############### Processing extra restraints explicitly defined

 No 'NOTE BUSTER_TARGET' cards or -target?? args found (used to read coordinates files for soft ncs to external)
 No 'NOTE BUSTER_OCCSUM'     cards found - so not using sum of occupancy restraints
 No 'NOTE BUSTER_DISTANCE'   cards found - so not setting up any additional atom-atom distance restraints
 No 'NOTE BUSTER_UTILANGLE'  cards found - so not setting up any additional restraints on any angle
 No 'NOTE BUSTER_UTILTOR'    cards found - so not setting up any additional sinusoidal restraints on torsions
 
 --- Have some similarity definitions 
     Similarity restraints on RMSD use superposition routines from: 
     Coutsias, E.A., Seok, C., Dill, K.A.(2004)
     "Using quaternions to calculate RMSD", J. Comput. Chem., 25:1849-1857 

 Have found    1 'NOTE BUSTER_SIM_DEFINE' cards 
     Maximum number of chains involved in any SIM group (excluding the template)=     1


 ########## Interpreting card: NOTE BUSTER_SIM_DEFINE ncsautoAB ncsautoABset B
     Number of atoms in template set=  2783
     Number of related chains (other than template) in SIM set=    1
     List of chains to be restrained (excluding template)=   B   
     Pairwise RMSD, XYZ in Angstroms, for SIM group: ncsautoAB
                 A         B   
     A         -----     ----- 
     B         0.699     ----- 
     Overall RMSD,XYZ for the SIM group is     0.699 Angstroms
     Pairwise rms deviation of [individual B's minus their set/chain average] in Angs**2
                 A         B         Bavg
     A         -----     -----    110.56
     B         42.66     -----     79.63
     Overall RMSD,B   for the SIM group is    42.663 Angs**2
     The number of common atoms for the above fits:
                 A         B   
     A         -----     ----- 
     B          2770     ----- 
     (compared to the template set that has    2783 atoms in it)
     List of atoms that are absent in one or more chains for this group: 
                                    A    B   
                    C01     501     +    -
                    N02     501     +    -
                    C03     501     +    -
                    C04     501     +    -
                    C05     501     +    -
                    C06     501     +    -
                    C07     501     +    -
                    N08     501     +    -
                    C09     501     +    -
                    C10     501     +    -
                    C11     501     +    -
                    N12     501     +    -
                    C13     501     +    -
     (key  +  atom present for this chain 
           -  atom absent  for this chain)
 transforms for each of the superpositions orthogonal matrix r11 r12 r13 r21 r22 r23 r31 r32 r33 tx ty tz (PDBSET syntax): 
TRANS_AB -0.874152 -0.484815 -0.028499 -0.484819  0.874585 -0.007231  0.028430  0.007496 -0.999568    33.983    33.043    14.746
 reverse transforms 
TRANS_BA -0.874152 -0.484819  0.028430 -0.484815  0.874585  0.007496 -0.028499 -0.007231 -0.999568    45.307   -12.534    15.947
 
 Setting up LSSR interatomic distance restraints
 Interpreting card: NOTE BUSTER_SIM_RESTRAIN_LSSR ncsautoAB 6.666
        setting up interatomic distance pair restraints with:
        Dmin=         0.00
        Dmax=         5.50
        sigma=        0.20
        Vmax=         3.00
        weight=       6.67
        number of distance pairs defined for this set is      35121
 Have not supplied either '-sim_swap_equiv' or '-sim_swap_equiv_plus' argument 
   so will not try swaping equivalent atoms to improve NCS and/or target agreement.
 
 NCS by LSSR outlier analysis
 -autoncs option automatic NCS prune routine -------- 
 Residues pruned out by gradient condition:
           A|54     A|131     A|135     A|140     A|148     A|150     A|151
          A|158     A|168     A|169     A|172     A|173     A|174     A|175
          A|181     A|182     A|184     A|185     A|186     A|187     A|189
          A|216     A|217     A|218     A|219     A|220     A|221     A|222
          A|237     A|250     A|253     A|255     A|256     A|259     A|260
          A|264     A|310     A|318     A|320     A|322     A|325     A|326
          A|327     A|328     A|329     A|344     A|345      B|54     B|131
          B|135     B|140     B|148     B|150     B|151     B|158     B|168
          B|169     B|172     B|173     B|174     B|175     B|181     B|182
          B|184     B|185     B|186     B|187     B|189     B|216     B|217
          B|218     B|219     B|220     B|221     B|222     B|237     B|250
          B|253     B|255     B|256     B|259     B|260     B|264     B|310
          B|318     B|320     B|322     B|325     B|326     B|327     B|328
          B|329     B|344     B|345
  
 Residues pruned out by function/max condition:
          A|149     A|168!    A|169!    A|170     A|171     A|172!    A|173!
          A|174!    A|187!    A|188     A|220!    A|259!    A|326!    A|346 
         A|ENDA     A|401     B|149     B|168!    B|169!    B|170     B|171 
          B|172!    B|173!    B|174!    B|187!    B|188     B|220!    B|259!
          B|326!    B|346    B|ENDA     B|401 
  
 Total number of residues for which LSSR restraints have been pruned out is      108 out of      700 participating
 Number of LSSR restraints pruned out is      7568 this is   21.5% of the total           35121
  
 -autoncs .Gelly cards suitable for adaptation for manual definition/pruning
           (note auto pruning is done in a slightly different way so exact results may differ slightly)
 -autoncs option .Gelly cards also written to file  auto_gelly_cards.txt
NOTE BUSTER_SET ncsautoXcld = Water    # card added - produced by -autoncs
NOTE BUSTER_SET ncsautoXcld = ncsautoXcld + { A|54 A|131 A|135 A|140 A|148 }    # card added - produced by -autoncs
NOTE BUSTER_SET ncsautoXcld = ncsautoXcld + { A|149 A|150 A|151 A|158 A|168 }    # card added - produced by -autoncs
NOTE BUSTER_SET ncsautoXcld = ncsautoXcld + { A|169 A|170 A|171 A|172 A|173 }    # card added - produced by -autoncs
NOTE BUSTER_SET ncsautoXcld = ncsautoXcld + { A|174 A|175 A|181 A|182 A|184 }    # card added - produced by -autoncs
NOTE BUSTER_SET ncsautoXcld = ncsautoXcld + { A|185 A|186 A|187 A|188 A|189 }    # card added - produced by -autoncs
NOTE BUSTER_SET ncsautoXcld = ncsautoXcld + { A|216 A|217 A|218 A|219 A|220 }    # card added - produced by -autoncs
NOTE BUSTER_SET ncsautoXcld = ncsautoXcld + { A|221 A|222 A|237 A|250 A|253 }    # card added - produced by -autoncs
NOTE BUSTER_SET ncsautoXcld = ncsautoXcld + { A|255 A|256 A|259 A|260 A|264 }    # card added - produced by -autoncs
NOTE BUSTER_SET ncsautoXcld = ncsautoXcld + { A|310 A|318 A|320 A|322 A|325 }    # card added - produced by -autoncs
NOTE BUSTER_SET ncsautoXcld = ncsautoXcld + { A|326 A|327 A|328 A|329 A|344 }    # card added - produced by -autoncs
NOTE BUSTER_SET ncsautoXcld = ncsautoXcld + { A|345 A|346 A|ENDA A|401 }    # card added - produced by -autoncs
NOTE BUSTER_SET ncsautoABset = Chain_A \ ncsautoXcld    # card added - produced by -autoncs
NOTE BUSTER_SIM_DEFINE ncsautoAB ncsautoABset B    # card added - produced by -autoncs
NOTE BUSTER_SIM_RESTRAIN_LSSR ncsautoAB 6.666    # card added - produced by -autoncs

 LSSR function contribution before pruning     59640.4
                             after pruning      7048.5
 Graph of which residues pruned from LSSR: ncs_pruned.mtv
 Graph of LSSR function outliers ncs_function_outliers.mtv
 Graph of LSSR gradient outliers ncs_gradient_outliers.mtv
 LSSR - analysis of distance difference distribution for NCS
         DeltaD less than 0.5 Angs:  proportion of population= 99.21% average DeltaD of subset     0.013 Angs
 Histogram of LSSR deltaD distribution ncs_histogram.mtv
 
 Interpreting card: NOTE BUSTER_TLS_SET tls1 { A|* }
 Interpreting card: NOTE BUSTER_TLS_SET tls2 { B|* }
 Interpreting card: NOTE BUSTER_TLS_SET tls1 { A|* }
 Interpreting card: NOTE BUSTER_TLS_T   tls1     -2.44720E-01 -1.33885E-01  3.49464E-01 -7.26320E-02  1.36549E-02 -3.31073E-02
 Interpreting card: NOTE BUSTER_TLS_L   tls1      2.21033E+00  2.43686E+00  1.90206E+00 -4.70482E-01 -1.42181E+00  6.26775E-01
 Interpreting card: NOTE BUSTER_TLS_S   tls1      1.26526E-01 -3.75851E-01  4.70780E-01 -3.29027E-01  1.72095E-01 -3.71904E-01  6.80978E-02 -3.47299E-01
 Interpreting card: NOTE BUSTER_TLS_O   tls1     -2.31731E+01 -8.40215E+01  1.10770E+01
 Interpreting card: NOTE BUSTER_TLS_SET tls2 { B|* }
 Interpreting card: NOTE BUSTER_TLS_T   tls2     -3.51018E-01 -2.65873E-01  4.05020E-01  2.48330E-02  3.11379E-02 -4.04722E-02
 Interpreting card: NOTE BUSTER_TLS_L   tls2      1.80307E+00  1.69393E+00  1.39140E+00  8.83117E-01 -1.11732E+00 -9.56109E-01
 Interpreting card: NOTE BUSTER_TLS_S   tls2     -1.42162E-02  9.70000E-02 -1.62700E-01  8.39808E-02  2.10687E-01  5.67053E-02 -9.55320E-02  7.37692E-02
 Interpreting card: NOTE BUSTER_TLS_O   tls2      2.22040E+01 -7.22942E+01  3.58164E+00
 Interpreting card: NOTE BUSTER_TLS_FIX tls1 ALL
 Interpreting card: NOTE BUSTER_TLS_FIX tls2 ALL


 TLS information. Total number of groups:     2


 Group   Natoms    FixRB   FixTLS   Group Tag    Group Spec
     1     2783      Y        Y     tls1    { A|* }
     2     2770      Y        Y     tls2    { B|* }
 
NOTE BUSTER_TLS_SET tls1 { A|* }
NOTE BUSTER_TLS_T   tls1     -2.44720E-01 -1.33885E-01  3.49464E-01 -7.26320E-02  1.36549E-02 -3.31073E-02
NOTE BUSTER_TLS_L   tls1      2.21033E+00  2.43686E+00  1.90206E+00 -4.70482E-01 -1.42181E+00  6.26775E-01
NOTE BUSTER_TLS_S   tls1      1.26526E-01 -3.75851E-01  4.70780E-01 -3.29027E-01  1.72095E-01 -3.71904E-01  6.80978E-02 -3.47299E-01
NOTE BUSTER_TLS_O   tls1     -2.31731E+01 -8.40215E+01  1.10770E+01
NOTE BUSTER_TLS_FIX tls1 ALL
 
NOTE BUSTER_TLS_SET tls2 { B|* }
NOTE BUSTER_TLS_T   tls2     -3.51018E-01 -2.65873E-01  4.05020E-01  2.48330E-02  3.11379E-02 -4.04722E-02
NOTE BUSTER_TLS_L   tls2      1.80307E+00  1.69393E+00  1.39140E+00  8.83117E-01 -1.11732E+00 -9.56109E-01
NOTE BUSTER_TLS_S   tls2     -1.42162E-02  9.70000E-02 -1.62700E-01  8.39808E-02  2.10687E-01  5.67053E-02 -9.55320E-02  7.37692E-02
NOTE BUSTER_TLS_O   tls2      2.22040E+01 -7.22942E+01  3.58164E+00
NOTE BUSTER_TLS_FIX tls2 ALL


 TLS: mmCIF group specification begin


#
loop_
_pdbx_refine_tls_group.id
_pdbx_refine_tls_group.refine_tls_id
_pdbx_refine_tls_group.beg_PDB_ins_code
_pdbx_refine_tls_group.beg_auth_asym_id
_pdbx_refine_tls_group.beg_auth_seq_id
_pdbx_refine_tls_group.end_PDB_ins_code
_pdbx_refine_tls_group.end_auth_asym_id
_pdbx_refine_tls_group.end_auth_seq_id
_pdbx_refine_tls_group.selection
_pdbx_refine_tls_group.selection_details
   1   1 . A   -1 . A  346 . '{ A|* }'
   2   1 . A  401 . A  501 . '{ A|* }'
   3   2 . B   -1 . B  346 . '{ B|* }'
   4   2 . B  401 . B  401 . '{ B|* }'
#


 TLS: mmCIF group specification end


 Interpreting card: NOTE BUSTER_ANISO_SET Empty
 Interpreting card: NOTE BUSTER_ANISO_WEIGHT SCA  16.0
 Interpreting card: NOTE BUSTER_ANISO_WEIGHT SPH   0.6    0.6
 Interpreting card: NOTE BUSTER_ANISO_WEIGHT SIM   7.8    4.8    2.6    0
 Interpreting card: NOTE BUSTER_ANISO_WEIGHT RIG  21.5   21.5    3.7    0


 After TLS and ANISO: Number of atoms with fixed/combined xyz =     0
 After TLS and ANISO: Number of atoms with fixed/combined B   =     0
 After TLS and ANISO: Number of atoms with fixed/combined OCC =  6519
 After TLS and ANISO: Number of atoms with fixed/combined BIJ =  6519
 After TLS and ANISO: Number of atoms in a TLS group          =  5553
                                     cf total number of atoms =  6519
 

 ############### Setup state vector

 No free_vect_ulim_xyz argument found: using default of  100.000
 No free_vect_llim_xyz argument found: using default of -100.000
 Number of parameters added for TLS description                 =       0
 Number of parameters to describe molecular conformation        =   26076

 ############### Setup free vector

 Number of free parameters to be adjusted in refinement   =   26076

 ############### Setting up own geometry


 No command line argument '-tnt_fn' so will use gelly to do geometry function calculations

 ============= Call TNT functions


 ============= Get TNT symmetry information

 
      Getting symmetry operators from TNT. 
          gelly will classify symmetry using pdb-like convention: 
               SYMOP   SYMMETRY
              NNNMMM   OPERATOR
                1555   X,Y,Z
                2555   -Y,X-Y,Z
                3555   -X+Y,-X,Z
                4555   -X,-Y,1/2+Z
                5555   Y,-X+Y,1/2+Z
                6555   X-Y,X,1/2+Z
          where NNN -> operator number and MMM -> translation vector
 

 ============= Own Gelly geometry/restraints setup


 ----------- Identify bond-types


    Setting up GEOMBONDREC_TYPE_HASH* to be able to map a given bond type (e.g.
    "single") to an internal integer (GBOND_TYPE=1) etc.

    Using default  GEOMBONDREC_TYPE_HASH_STR='SING* 1 DOUB* 2 TRIP* 3 AROM* 4 DELO* 4 METAL* 5':
    to override default use a NOTE BUSTER_GEOMBONDREC_TYPE_HASH_STR gelly card.

 ----------- Classify torsion into peptide omega and other


 ----------- Setup of non-bonded terms

 Ideal-distance contact term setup:
    Restraint function constants (normally these values are from NOTE BUSTER_IDEAL_CONTACT_* cards)
    Sigma for general contacts      0.200 Angs
    Sigma for 1-4 contacts          0.100 Angs (contacts across a torsion)
    Sigma for 1-5 contacts          0.150 Angs
    Sigma for 1-N contacts          0.200 Angs
    Sigma for HBOND pairs           0.100 Angs (contacts between HB_types A and D or B's)
    Contact distance increment for each atom in HBOND          -0.150 Angs 
    Contact distance increment for H-bond acceptor              0.100 Angs 
    Contact distance increment for 1-4 with an O atom          -0.050 Angs 
    Contact distance increment for 1-4 with an N atom          -0.180 Angs 
    Contact distance increment for 1-4 with an C atom          -0.370 Angs 
    Contact distance increment for 1-4 with an S atom          -0.150 Angs 
    Contact distance increment for 1-4 with an P atom          -0.150 Angs 
    Contact distance increment for 1-4 with any other atom     -0.150 Angs 
    All atoms matched for CCP4 atom type.
    Number of CCP4 atom types used is         23 (H-bound and not H-bound counted separately)

 Setting up short/ideal-distance contacts
   Using gelly special position handling. (For TNT instead rerun with argument '-special_tnt')

   No command line argument '-special_all' specified. We will auto-generate
   EXCLUDE cards only for atom-atom contacts across symmetry (i.e. on special
   position) when this involves a contact within a residue to itself.

   Have set up non-bonded exclusions - 1-3 contacts
   Atom A|27:CA         makes the most 1-3 contacts:  6

   Have set up non-bonded exclusions - 1-4 contacts
   Atom A|27:N          makes the most 1-4 contacts:  7

   Have set up non-bonded exclusions - 1-5 contacts
   Atom A|28:C.A        makes the most 1-5 contacts: 10

 ############################################################################################################
 ###################################################### setting up contact terms
 ############################################################################################################


 ============= Call Gelly versions of TNT functions

 WARNING: 1-4 contact without 1-(2,3)-4 data for A|401:C2(CIT)        and A|401:O6(CIT)        X-X
 WARNING: 1-4 contact without 1-(2,3)-4 data for A|401:O7(CIT)        and A|401:C5(CIT)        X-X
 WARNING: 1-4 contact without 1-(2,3)-4 data for A|401:O7(CIT)        and A|401:O5(CIT)        X-X
 WARNING: 1-4 contact without 1-(2,3)-4 data for A|401:C4(CIT)        and A|401:O6(CIT)        X-X
 WARNING: 1-4 contact without 1-(2,3)-4 data for A|501:C01(LIG)       and A|501:N08(LIG)       X-X
 WARNING: 1-4 contact without 1-(2,3)-4 data for A|501:N02(LIG)       and A|501:C10(LIG)       X-X
 WARNING: 1-4 contact without 1-(2,3)-4 data for B|401:C1(CIT)        and B|401:C4(CIT)        X-X
 WARNING: 1-4 contact without 1-(2,3)-4 data for B|401:C2(CIT)        and B|401:O6(CIT)        X-X
 WARNING: 1-4 contact without 1-(2,3)-4 data for B|401:O7(CIT)        and B|401:O5(CIT)        X-X
 WARNING: 1-4 contact without 1-(2,3)-4 data for B|401:C4(CIT)        and B|401:O6(CIT)        X-X

       TERM      weight      number      TNT_fn    Gelly_fn        diff
       BOND       2.000        5668    1815.696    1815.696      -0.000
       ANGL       2.000        7691    5594.410    5594.410       0.000
       IMPR       0.000           0       0.000       0.000       0.000
       TORS       2.000        1973    3492.726    3492.726      -0.000
       TRIG       2.000           0       0.000       0.000       0.000
       PLAN       5.000         967    2282.501    2282.501       0.000
       CONT       5.000           0     138.212       0.000     138.212
       BCOR      10.000        5630    7970.525   12971.040   -5000.515


 ============= Call Gelly geometry functions

   Switching torsion functional form to sinusoidal rather than split harmonic functional.
      if you want this not to happen then rerun with '-torsharm'
      Weight for sinusoidal torsion=     2.000
   BCOR function/gradient will be split between H and non-H atoms (as for any atom pair). Use '-bcorrel_all_in_hydrogens' to change this.

 ----------- Fix B-correlation restraints

 
 Checking for bonds that do not have a bcorrel restraint defined
 We will wipe all existing BCORrels if OCC=0.0 atom(s) is/are involved. This can be' switched off via the '-bcorrel_nowipe_zeroocc' flag.
 We will wipe all existing BCORrels if only non-hydrogen atom(s) with fixed B(iso) are involved. This can be switched off via the '-bcorrel_nowipe_fixb_nonhydrogens' flag.
 We will keep all existing BCORrels if hydrogen atom(s) with fixed B(iso) is/are involved. This can be switched off via the '-bcorrel_wipe_fixb_hydrogens' flag.
 No -bcorrel_missing_sigma found. So will set sigma to     2.5 Ang**2 for any bond with no existing BCORrel
 No -bcorrel_missing_sigma_hydrogens found. So will set sigma to     1.0 Ang**2 for any bond with no existing BCORrel involving hydrogens

 No active BCORels found involving OCC=0.0 atom(s)


 No active BCORels found involving fixed-B (only) non-hydrogen atom(s)

    add a default BCOR for bond A|401:C1(CIT) A|401:C2(CIT)
    add a default BCOR for bond A|401:C1(CIT) A|401:O1(CIT)
    add a default BCOR for bond A|401:C1(CIT) A|401:O2(CIT)
    add a default BCOR for bond A|401:C2(CIT) A|401:C3(CIT)
    add a default BCOR for bond A|401:C3(CIT) A|401:C4(CIT)
    add a default BCOR for bond A|401:C3(CIT) A|401:C6(CIT)
    add a default BCOR for bond A|401:C3(CIT) A|401:O7(CIT)
    add a default BCOR for bond A|401:C4(CIT) A|401:C5(CIT)
    add a default BCOR for bond A|401:C5(CIT) A|401:O3(CIT)
    add a default BCOR for bond A|401:C5(CIT) A|401:O4(CIT)
    add a default BCOR for bond A|401:C6(CIT) A|401:O5(CIT)
    add a default BCOR for bond A|401:C6(CIT) A|401:O6(CIT)
    add a default BCOR for bond A|501:C01(LIG) A|501:N02(LIG)
    add a default BCOR for bond A|501:C01(LIG) A|501:C09(LIG)
    add a default BCOR for bond A|501:N02(LIG) A|501:C03(LIG)
    add a default BCOR for bond A|501:C03(LIG) A|501:C04(LIG)
    add a default BCOR for bond A|501:C03(LIG) A|501:N08(LIG)
    add a default BCOR for bond A|501:C04(LIG) A|501:C05(LIG)
    add a default BCOR for bond A|501:C05(LIG) A|501:C06(LIG)
    add a default BCOR for bond A|501:C06(LIG) A|501:C07(LIG)
    add a default BCOR for bond A|501:C07(LIG) A|501:N08(LIG)
    add a default BCOR for bond A|501:C09(LIG) A|501:C10(LIG)
    add a default BCOR for bond A|501:C09(LIG) A|501:C13(LIG)
    add a default BCOR for bond A|501:C10(LIG) A|501:C11(LIG)
    add a default BCOR for bond A|501:C11(LIG) A|501:N12(LIG)
    add a default BCOR for bond A|501:N12(LIG) A|501:C13(LIG)
    add a default BCOR for bond B|401:C1(CIT) B|401:C2(CIT)
    add a default BCOR for bond B|401:C1(CIT) B|401:O1(CIT)
    add a default BCOR for bond B|401:C1(CIT) B|401:O2(CIT)
    add a default BCOR for bond B|401:C2(CIT) B|401:C3(CIT)
    add a default BCOR for bond B|401:C3(CIT) B|401:C4(CIT)
    add a default BCOR for bond B|401:C3(CIT) B|401:C6(CIT)
    add a default BCOR for bond B|401:C3(CIT) B|401:O7(CIT)
    add a default BCOR for bond B|401:C4(CIT) B|401:C5(CIT)
    add a default BCOR for bond B|401:C5(CIT) B|401:O3(CIT)
    add a default BCOR for bond B|401:C5(CIT) B|401:O4(CIT)
    add a default BCOR for bond B|401:C6(CIT) B|401:O5(CIT)
    add a default BCOR for bond B|401:C6(CIT) B|401:O6(CIT)

 Have added a total of        38 BCORrels for bonds which do not already have a BCORrel


 No bonds (for potential BCORrel addition) were skipped due to both atoms having constant/fixed B(iso)


 ----------- Deactivate hydrogen-free planes if hydrogen atoms present

 A total of       0 planes have been deactivated because they are duplicated hydrogen-free and with-hydrogen forms.

 ----------- Handle peptide omega angle(s)

      Torsion angles split into peptide omega and other
         Number of peptide omega angles is       698
         Number of other torsion is             1275
      Peptide omega angles are monitored rather than directly restrained (PLANE used instead).

 ----------- Fix cis-PRO

 Now apply the fix to get correct restraints for cis PRO residues
   Have got      11 bonds records and      26 angles
   from GEOMETRY cards for the special PROC residue
   Weight for ideal-distance contact term=     4.000

 ----------- Prepare SSBOND/LINK for output PDB file


 ----------- Prepare for QM


 ----------- Prepare symmetry in utility distance restraints


 ############### Setting up Rhofit contacts (if needed)

 No ligand-(fixed protein) contact term will be used

 ############### Working out initial function and gradient


   MaxLik Scaling Cycle 0002
 
 Initial functional value = 4.080538E+05 rms gradient=      75.3699
 X-ray contribution to function value = 3.629060E+05
 Geometry contribution (inc ncs)      =      45147.8
 Geometry function summary
     term                           number     weight       rms        GooF      function     fn/numb
     ================================================================================================
     BOND bond lengths (angs)       5668       2.000      0.0080       0.400      1815.70       0.320
     ANGL bond angles (degs)        7691       2.000        1.01       0.603      5594.41       0.727
     TORS split-harmonic tors       1275       0.000       18.62       1.170         0.00       0.000
     SINTOR sinusoidal tors         1275       2.000       18.62                  2619.49       2.054
     PLAN general plane (angs)       967       5.000      0.0137       0.687      2282.50       2.360
     BCORrelations (angs^2)         5668      10.000       1.341       0.479     12992.40       2.292
     CONT (bad contacts)               0       5.000                                 0.00
     IDEAL(ideal-dist contact)      5585       4.000                             12749.19
     CHIRAL (gelly semiharmon)       740       5.000                                45.69
     SIM similarity restraint for NCS and/or TARGET                               7048.47
     OCCSUM restraint on sum of occupanies                                           0.00       as none defined
     DISTAN utility restraints interatomic distance                                  0.00       as none defined
     UTILANGLE utility angle restraints                                              0.00       as none defined
     UTILTOR utility restraints on torsion angles                                    0.00       as none defined
     Peptide omega torsion angles                           2.83 degs - monitor only
 
 WARNING: Have |delta/sigma| deviations > 5.0 sigma. Number of outliers for each term: 
 WARNING:         2 bond angles.  Worst is     5.2 sigs   134.58 degs A|28:C.A(ASN)=A|29:N(ASN)=A|29:CA(ASN)

 Weighted rms      0.0080         1.012  (temporary rms bond and angle for BUSTER to pick up!)
 Weighted intra-residual rms(bond) for StdProtein (excluding H)      0.0078
 Weighted intra-residual rms(bond) for Buffer     (excluding H)      0.0030

 ############### Running initial check


 Initial Geometry Sanity Check

 Check for duplicate bonds: no duplicates found
 
 Check for duplicate or missing bond angles: all are OK
 Check for bond between an atom and itself not involving symmetry:  all are OK
   There are no bad contacts where Delta/sigma is greater than     6.5
   Worst contact dist=   3.029 std=   3.802 sigma=   0.206 abs(Delta/sigma)=    3.754 ats A|104:CE (MET) B|23:CB (PRO)
   It is likely that the geometry libraries, sequence info and links match the input molecule
 GEOMETRY CHECK OVERALL:  PASS


 ############### Creating geometry/screen report


 ############### Option "tao" - run Tao optimizer

 TAO: Toolkit for Advanced Optimization. Reference: 
     Munson, T., Sarich, J., Wild, S., Benson, S., McInnes, L.C. (2018)
     "TAO User Manual (Revision 3.9)", Mathematics and Computer Science
     Division, Argonne National Laboratory
     ANL/MCS-TM-322, http://www.mcs.anl.gov/petsc/
 
 Have found -keeppdb flag so will write out a .pdb file every 1000000 its during opt
 Will apply TAO optimization 
 Maximum number of iterations      100
 Convergence test on Grms       4.0000 (-glim)
 Limit on rms displacement to original position (rmsD2init): NONE test inactive (-dlim)

    analysing argument no 0 : -autoncs with j=0
    analysing argument no 1 : -hydrogens_input_occ2zero with j=0
    analysing argument no 2 : -hydrogens_output_occ2heavy with j=0
    analysing argument no 3 : -screen with j=0
    analysing argument no 4 : 100 with j=0
    analysing argument no 5 : -screen_sigma with j=0
    analysing argument no 6 : 3.0 with j=0
    analysing argument no 7 : -keeppdb with j=0
    analysing argument no 8 : 1000000 with j=0
    analysing argument no 9 : -taomonitor_verbose with j=0
    found argument starting with "-tao" : -taomonitor_verbose
    analysing argument no 10 : 2 with j=1
    found value after -tao/-options_/-viewer_ argument : "2"
 PETSC/TAO: setting option "-taomonitor_verbose" = "2"


   MaxLik Scaling Cycle 0003
   Iters     Tot_Funct    Xray_Funct    Geom_Funct          Grms     rmsD2init     maxD2init             R         Rfree           LLG       LLGfree     rms(bond)    rms(angle)       Opttag_H
   Iters             K   B_IMPF_FRAG        K_MISS   B_TEMP_MISS   K_IMPF_MISS   B_IMPF_MISS        K_SOLV   B_TEMP_SOLV   K_IMPF_SOLV   B_IMPF_SOLV        B_TEMP     B_TEMP_11     B_TEMP_12     B_TEMP_22     B_TEMP_13     B_TEMP_23     B_TEMP_33      K_WILSON      B_WILSON      FGCYCLES       Scatag_H
   Iters          BOND         ANGLE      IMPROPER       TORSION    SINTORSION      TRIGONAL        PLANAR  BCORRELATION       CONTACT     IDEALDIST        CHIRAL    SIMILARITY       OCCSSUM  UTILDISTANCE     UTILANGLE   UTILTORSION        RHOFIT        ADP-01        ADP-02        ADP-03        ADP-04        ADP-05        ADP-06        ADP-07        ADP-08        ADP-09        ADP-10      RHOFITPC            QM       Funtag_H
       0  4.079046E+05  3.629040E+05       45000.6       68.1175  0.000000E+00  0.000000E+00      0.185540      0.241357  0.000000E+00  0.000000E+00    0.00802436       1.01215       Opttag_D
       0       81.9114       1.05842       1.00000  0.000000E+00       1.00000  0.000000E+00      0.943349       103.159       1.00000       36.8811  0.000000E+00      -1.39383  0.000000E+00      -1.39383  0.000000E+00  0.000000E+00       2.78767       82.3604    -0.0687431        2       Scatag_D
       0       1815.70       5594.41  0.000000E+00  0.000000E+00       2619.49  0.000000E+00       2282.50       12845.2  0.000000E+00       12749.2       45.6919       7048.47 -1.000000E+10 -1.000000E+10 -1.000000E+10 -1.000000E+10 -1.000000E+10  0.000000E+00  0.000000E+00  0.000000E+00  0.000000E+00  0.000000E+00  0.000000E+00  0.000000E+00  0.000000E+00  0.000000E+00  0.000000E+00  0.000000E+00  0.000000E+00       Funtag_D
   Geometry Restraint Deviations SCREEN Information

 

   MaxLik Scaling Cycle 0004

   MaxLik Scaling Cycle 0005

   MaxLik Scaling Cycle 0006

   Wilson plot

   Statistics in resolution bins after refinement cycle 0001
```


|  |  |
| --- | --- |
|  | - Rxpct factors plot- Rxpct factors table- <Log-Lik Gain>- Ln Sigma A- Correlation Coefficients |


```
   Iters     Tot_Funct    Xray_Funct    Geom_Funct          Grms     rmsD2init     maxD2init             R         Rfree           LLG       LLGfree     rms(bond)    rms(angle)
   Iters             K   B_IMPF_FRAG        K_MISS   B_TEMP_MISS   K_IMPF_MISS   B_IMPF_MISS        K_SOLV   B_TEMP_SOLV   K_IMPF_SOLV   B_IMPF_SOLV        B_TEMP     B_TEMP_11     B_TEMP_12     B_TEMP_22     B_TEMP_13     B_TEMP_23     B_TEMP_33      K_WILSON      B_WILSON      FGCYCLES
   Iters          BOND         ANGLE      IMPROPER       TORSION    SINTORSION      TRIGONAL        PLANAR  BCORRELATION       CONTACT     IDEALDIST        CHIRAL    SIMILARITY       OCCSSUM  UTILDISTANCE     UTILANGLE   UTILTORSION        RHOFIT        ADP-01        ADP-02        ADP-03        ADP-04        ADP-05        ADP-06        ADP-07        ADP-08        ADP-09        ADP-10      RHOFITPC            QM
       1  4.068393E+05  3.628372E+05       44002.1       52.1014  4.233612E-04    0.00371741      0.185514      0.241584  2.458557E-04 -9.842900E-04    0.00803740       1.01242       Opttag_D
       1       81.8947       1.05539       1.00000  0.000000E+00       1.00000  0.000000E+00      0.943371       103.079       1.00000       36.8628  0.000000E+00      -1.38733  0.000000E+00      -1.38733  0.000000E+00  0.000000E+00       2.77466       82.3604    -0.0687431        3       Scatag_D
       1       1821.60       5597.37  0.000000E+00  0.000000E+00       2619.42  0.000000E+00       2282.80       11854.1  0.000000E+00       12751.1       45.6980       7029.96 -1.000000E+10 -1.000000E+10 -1.000000E+10 -1.000000E+10 -1.000000E+10  0.000000E+00  0.000000E+00  0.000000E+00  0.000000E+00  0.000000E+00  0.000000E+00  0.000000E+00  0.000000E+00  0.000000E+00  0.000000E+00  0.000000E+00  0.000000E+00       Funtag_D
   Geometry Restraint Deviations SCREEN Information

 

   MaxLik Scaling Cycle 0007

   Wilson plot

   Statistics in resolution bins after refinement cycle 0002
```


|  |  |
| --- | --- |
|  | - Rxpct factors plot- Rxpct factors table- <Log-Lik Gain>- Ln Sigma A- Correlation Coefficients |


```
   Iters     Tot_Funct    Xray_Funct    Geom_Funct          Grms     rmsD2init     maxD2init             R         Rfree           LLG       LLGfree     rms(bond)    rms(angle)
   Iters             K   B_IMPF_FRAG        K_MISS   B_TEMP_MISS   K_IMPF_MISS   B_IMPF_MISS        K_SOLV   B_TEMP_SOLV   K_IMPF_SOLV   B_IMPF_SOLV        B_TEMP     B_TEMP_11     B_TEMP_12     B_TEMP_22     B_TEMP_13     B_TEMP_23     B_TEMP_33      K_WILSON      B_WILSON      FGCYCLES
   Iters          BOND         ANGLE      IMPROPER       TORSION    SINTORSION      TRIGONAL        PLANAR  BCORRELATION       CONTACT     IDEALDIST        CHIRAL    SIMILARITY       OCCSSUM  UTILDISTANCE     UTILANGLE   UTILTORSION        RHOFIT        ADP-01        ADP-02        ADP-03        ADP-04        ADP-05        ADP-06        ADP-07        ADP-08        ADP-09        ADP-10      RHOFITPC            QM
       2  4.063812E+05  3.627548E+05       43626.4       58.5677    0.00101072     0.0102667      0.185484      0.241569  4.718926E-04   -0.00116564    0.00807676       1.01626       Opttag_D
       2       81.8700       1.05145       1.00000  0.000000E+00       1.00000  0.000000E+00      0.943349       103.000       1.00000       36.8283  0.000000E+00      -1.37855  0.000000E+00      -1.37855  0.000000E+00  0.000000E+00       2.75710       82.3604    -0.0687431        1       Scatag_D
       2       1839.49       5639.93  0.000000E+00  0.000000E+00       2619.10  0.000000E+00       2284.30       11437.0  0.000000E+00       12754.2       45.7997       7006.67 -1.000000E+10 -1.000000E+10 -1.000000E+10 -1.000000E+10 -1.000000E+10  0.000000E+00  0.000000E+00  0.000000E+00  0.000000E+00  0.000000E+00  0.000000E+00  0.000000E+00  0.000000E+00  0.000000E+00  0.000000E+00  0.000000E+00  0.000000E+00       Funtag_D
   Geometry Restraint Deviations SCREEN Information

 
       3  4.060749E+05  3.627126E+05       43362.3       41.3952    0.00129869     0.0141362      0.185470      0.241571  5.877567E-04   -0.00124811    0.00808839       1.01382       Opttag_D
       3       81.8564       1.04941       1.00000  0.000000E+00       1.00000  0.000000E+00      0.943325       102.966       1.00000       36.8098  0.000000E+00      -1.37410  0.000000E+00      -1.37410  0.000000E+00  0.000000E+00       2.74821       82.3604    -0.0687431        1       Scatag_D
       3       1844.79       5612.87  0.000000E+00  0.000000E+00       2619.16  0.000000E+00       2285.67       11201.7  0.000000E+00       12756.7       45.8121       6995.61 -1.000000E+10 -1.000000E+10 -1.000000E+10 -1.000000E+10 -1.000000E+10  0.000000E+00  0.000000E+00  0.000000E+00  0.000000E+00  0.000000E+00  0.000000E+00  0.000000E+00  0.000000E+00  0.000000E+00  0.000000E+00  0.000000E+00  0.000000E+00       Funtag_D
       4  4.058423E+05  3.626580E+05       43184.3       33.9801    0.00172448     0.0196934      0.185448      0.241578  7.376688E-04   -0.00136270    0.00810984       1.01409       Opttag_D
       4       81.8373       1.04676       1.00000  0.000000E+00       1.00000  0.000000E+00      0.943288       102.921       1.00000       36.7838  0.000000E+00      -1.36781  0.000000E+00      -1.36781  0.000000E+00  0.000000E+00       2.73563       82.3604    -0.0687431        1       Scatag_D
       4       1854.59       5615.79  0.000000E+00  0.000000E+00       2619.18  0.000000E+00       2288.03       11019.2  0.000000E+00       12760.0       45.7852       6981.71 -1.000000E+10 -1.000000E+10 -1.000000E+10 -1.000000E+10 -1.000000E+10  0.000000E+00  0.000000E+00  0.000000E+00  0.000000E+00  0.000000E+00  0.000000E+00  0.000000E+00  0.000000E+00  0.000000E+00  0.000000E+00  0.000000E+00  0.000000E+00       Funtag_D
       5  4.056194E+05  3.625778E+05       43041.6       36.9380    0.00244758     0.0294256      0.185417      0.241596  9.576075E-04   -0.00155576    0.00815504       1.01616       Opttag_D
       5       81.8053       1.04267       1.00000  0.000000E+00       1.00000  0.000000E+00      0.943224       102.847       1.00000       36.7422  0.000000E+00      -1.35719  0.000000E+00      -1.35719  0.000000E+00  0.000000E+00       2.71437       82.3604    -0.0687431        1       Scatag_D
       5       1875.32       5638.75  0.000000E+00  0.000000E+00       2619.10  0.000000E+00       2292.90       10841.9  0.000000E+00       12765.7       45.6751       6962.18 -1.000000E+10 -1.000000E+10 -1.000000E+10 -1.000000E+10 -1.000000E+10  0.000000E+00  0.000000E+00  0.000000E+00  0.000000E+00  0.000000E+00  0.000000E+00  0.000000E+00  0.000000E+00  0.000000E+00  0.000000E+00  0.000000E+00  0.000000E+00       Funtag_D
       6  4.054396E+05  3.624917E+05       42947.9       43.1464    0.00342176     0.0433071      0.185391      0.241638    0.00119411   -0.00183033    0.00820310       1.01946       Opttag_D
       6       81.7622       1.03781       1.00000  0.000000E+00       1.00000  0.000000E+00      0.943126       102.757       1.00000       36.6896  0.000000E+00      -1.34342  0.000000E+00      -1.34342  0.000000E+00  0.000000E+00       2.68684       82.3604    -0.0687431        1       Scatag_D
       6       1897.49       5675.44  0.000000E+00  0.000000E+00       2619.12  0.000000E+00       2300.72       10694.0  0.000000E+00       12774.3       45.5240       6941.32 -1.000000E+10 -1.000000E+10 -1.000000E+10 -1.000000E+10 -1.000000E+10  0.000000E+00  0.000000E+00  0.000000E+00  0.000000E+00  0.000000E+00  0.000000E+00  0.000000E+00  0.000000E+00  0.000000E+00  0.000000E+00  0.000000E+00  0.000000E+00       Funtag_D
       7  4.052803E+05  3.624711E+05       42809.2       28.5197    0.00374050     0.0485487      0.185387      0.241659    0.00125062   -0.00192523    0.00819863       1.01667       Opttag_D
       7       81.7467       1.03641       1.00000  0.000000E+00       1.00000  0.000000E+00      0.943083       102.732       1.00000       36.6727  0.000000E+00      -1.33898  0.000000E+00      -1.33898  0.000000E+00  0.000000E+00       2.67796       82.3604    -0.0687431        1       Scatag_D
       7       1895.42       5644.44  0.000000E+00  0.000000E+00       2619.13  0.000000E+00       2303.68       10590.7  0.000000E+00       12776.8       45.4648       6933.49 -1.000000E+10 -1.000000E+10 -1.000000E+10 -1.000000E+10 -1.000000E+10  0.000000E+00  0.000000E+00  0.000000E+00  0.000000E+00  0.000000E+00  0.000000E+00  0.000000E+00  0.000000E+00  0.000000E+00  0.000000E+00  0.000000E+00  0.000000E+00       Funtag_D
       8  4.051444E+05  3.624510E+05       42693.4       26.3931    0.00418209     0.0558969      0.185388      0.241696    0.00130581   -0.00205407    0.00820164       1.01717       Opttag_D
       8       81.7255       1.03475       1.00000  0.000000E+00       1.00000  0.000000E+00      0.943019       102.703       1.00000       36.6507  0.000000E+00      -1.33332  0.000000E+00      -1.33332  0.000000E+00  0.000000E+00       2.66664       82.3604    -0.0687431        1       Scatag_D
       8       1896.81       5650.04  0.000000E+00  0.000000E+00       2619.11  0.000000E+00       2307.64       10471.5  0.000000E+00       12779.8       45.4271       6923.10 -1.000000E+10 -1.000000E+10 -1.000000E+10 -1.000000E+10 -1.000000E+10  0.000000E+00  0.000000E+00  0.000000E+00  0.000000E+00  0.000000E+00  0.000000E+00  0.000000E+00  0.000000E+00  0.000000E+00  0.000000E+00  0.000000E+00  0.000000E+00       Funtag_D
       9  4.050188E+05  3.624211E+05       42597.7       29.9879    0.00488488     0.0671531      0.185384      0.241750    0.00138773   -0.00223059    0.00821213       1.01855       Opttag_D
       9       81.6929       1.03223       1.00000  0.000000E+00       1.00000  0.000000E+00      0.942929       102.655       1.00000       36.6169  0.000000E+00      -1.32444  0.000000E+00      -1.32444  0.000000E+00  0.000000E+00       2.64887       82.3604    -0.0687431        1       Scatag_D
       9       1901.67       5665.33  0.000000E+00  0.000000E+00       2619.15  0.000000E+00       2313.44       10357.1  0.000000E+00       12785.9       45.4586       6909.67 -1.000000E+10 -1.000000E+10 -1.000000E+10 -1.000000E+10 -1.000000E+10  0.000000E+00  0.000000E+00  0.000000E+00  0.000000E+00  0.000000E+00  0.000000E+00  0.000000E+00  0.000000E+00  0.000000E+00  0.000000E+00  0.000000E+00  0.000000E+00       Funtag_D
      10  4.048732E+05  3.623872E+05       42485.9       31.8480    0.00610327     0.0863672      0.185391      0.241842    0.00148072   -0.00250455    0.00822614       1.01996       Opttag_D
      10       81.6384       1.02849       1.00000  0.000000E+00       1.00000  0.000000E+00      0.942773       102.576       1.00000       36.5618  0.000000E+00      -1.30958  0.000000E+00      -1.30958  0.000000E+00  0.000000E+00       2.61915       82.3604    -0.0687431        1       Scatag_D
      10       1908.16       5681.09  0.000000E+00  0.000000E+00       2619.16  0.000000E+00       2322.97       10223.6  0.000000E+00       12794.7       45.5918       6890.66 -1.000000E+10 -1.000000E+10 -1.000000E+10 -1.000000E+10 -1.000000E+10  0.000000E+00  0.000000E+00  0.000000E+00  0.000000E+00  0.000000E+00  0.000000E+00  0.000000E+00  0.000000E+00  0.000000E+00  0.000000E+00  0.000000E+00  0.000000E+00       Funtag_D
      11  4.047499E+05  3.623720E+05       42377.9       25.1617    0.00674335     0.0961795      0.185395      0.241886    0.00152250   -0.00262856    0.00823328       1.01959       Opttag_D
      11       81.6101       1.02663       1.00000  0.000000E+00       1.00000  0.000000E+00      0.942701       102.530       1.00000       36.5319  0.000000E+00      -1.30160  0.000000E+00      -1.30160  0.000000E+00  0.000000E+00       2.60319       82.3604    -0.0687431        1       Scatag_D
      11       1911.47       5676.93  0.000000E+00  0.000000E+00       2619.13  0.000000E+00       2326.94       10115.1  0.000000E+00       12800.1       45.6842       6882.54 -1.000000E+10 -1.000000E+10 -1.000000E+10 -1.000000E+10 -1.000000E+10  0.000000E+00  0.000000E+00  0.000000E+00  0.000000E+00  0.000000E+00  0.000000E+00  0.000000E+00  0.000000E+00  0.000000E+00  0.000000E+00  0.000000E+00  0.000000E+00       Funtag_D
      12  4.046310E+05  3.623557E+05       42275.3       23.5490    0.00747249      0.107019      0.185404      0.241924    0.00156742   -0.00274978    0.00824163       1.02025       Opttag_D
      12       81.5781       1.02458       1.00000  0.000000E+00       1.00000  0.000000E+00      0.942620       102.478       1.00000       36.4967  0.000000E+00      -1.29263  0.000000E+00      -1.29263  0.000000E+00  0.000000E+00       2.58525       82.3604    -0.0687431        1       Scatag_D
      12       1915.35       5684.26  0.000000E+00  0.000000E+00       2619.14  0.000000E+00       2330.36       10000.2  0.000000E+00       12806.2       45.7963       6874.02 -1.000000E+10 -1.000000E+10 -1.000000E+10 -1.000000E+10 -1.000000E+10  0.000000E+00  0.000000E+00  0.000000E+00  0.000000E+00  0.000000E+00  0.000000E+00  0.000000E+00  0.000000E+00  0.000000E+00  0.000000E+00  0.000000E+00  0.000000E+00       Funtag_D
      13  4.045390E+05  3.623375E+05       42201.5       26.2165    0.00833300      0.119246      0.185412      0.241956    0.00161722   -0.00285688    0.00825251       1.02140       Opttag_D
      13       81.5415       1.02218       1.00000  0.000000E+00       1.00000  0.000000E+00      0.942528       102.415       1.00000       36.4565  0.000000E+00      -1.28252  0.000000E+00      -1.28252  0.000000E+00  0.000000E+00       2.56504       82.3604    -0.0687431        1       Scatag_D
      13       1920.41       5697.04  0.000000E+00  0.000000E+00       2619.15  0.000000E+00       2333.80       9906.46  0.000000E+00       12812.8       45.8771       6866.00 -1.000000E+10 -1.000000E+10 -1.000000E+10 -1.000000E+10 -1.000000E+10  0.000000E+00  0.000000E+00  0.000000E+00  0.000000E+00  0.000000E+00  0.000000E+00  0.000000E+00  0.000000E+00  0.000000E+00  0.000000E+00  0.000000E+00  0.000000E+00       Funtag_D
      14  4.044415E+05  3.623195E+05       42121.9       20.5302    0.00926889      0.131874      0.185425      0.241984    0.00166662   -0.00295094    0.00826402       1.02099       Opttag_D
      14       81.5032       1.01966       1.00000  0.000000E+00       1.00000  0.000000E+00      0.942429       102.349       1.00000       36.4138  0.000000E+00      -1.27220  0.000000E+00      -1.27220  0.000000E+00  0.000000E+00       2.54441       82.3604    -0.0687431        1       Scatag_D
      14       1925.77       5692.48  0.000000E+00  0.000000E+00       2619.13  0.000000E+00       2336.94       9823.03  0.000000E+00       12819.6       45.9342       6859.02 -1.000000E+10 -1.000000E+10 -1.000000E+10 -1.000000E+10 -1.000000E+10  0.000000E+00  0.000000E+00  0.000000E+00  0.000000E+00  0.000000E+00  0.000000E+00  0.000000E+00  0.000000E+00  0.000000E+00  0.000000E+00  0.000000E+00  0.000000E+00       Funtag_D
      15  4.043541E+05  3.623007E+05       42053.4       19.5456     0.0101690      0.143160      0.185438      0.242004    0.00171845   -0.00303171    0.00827131       1.02186       Opttag_D
      15       81.4680       1.01720       1.00000  0.000000E+00       1.00000  0.000000E+00      0.942333       102.287       1.00000       36.3751  0.000000E+00      -1.26320  0.000000E+00      -1.26320  0.000000E+00  0.000000E+00       2.52639       82.3604    -0.0687431        1       Scatag_D
      15       1929.18       5702.27  0.000000E+00  0.000000E+00       2619.23  0.000000E+00       2339.52       9736.21  0.000000E+00       12827.2       45.9912       6853.78 -1.000000E+10 -1.000000E+10 -1.000000E+10 -1.000000E+10 -1.000000E+10  0.000000E+00  0.000000E+00  0.000000E+00  0.000000E+00  0.000000E+00  0.000000E+00  0.000000E+00  0.000000E+00  0.000000E+00  0.000000E+00  0.000000E+00  0.000000E+00       Funtag_D
      16  4.042722E+05  3.622811E+05       41991.1       19.8354     0.0111572      0.154558      0.185455      0.242024    0.00177221   -0.00314014    0.00828238       1.02253       Opttag_D
      16       81.4313       1.01458       1.00000  0.000000E+00       1.00000  0.000000E+00      0.942228       102.222       1.00000       36.3346  0.000000E+00      -1.25402  0.000000E+00      -1.25402  0.000000E+00  0.000000E+00       2.50805       82.3604    -0.0687431        1       Scatag_D
      16       1934.34       5709.73  0.000000E+00  0.000000E+00       2619.31  0.000000E+00       2341.89       9655.98  0.000000E+00       12834.9       46.0882       6848.91 -1.000000E+10 -1.000000E+10 -1.000000E+10 -1.000000E+10 -1.000000E+10  0.000000E+00  0.000000E+00  0.000000E+00  0.000000E+00  0.000000E+00  0.000000E+00  0.000000E+00  0.000000E+00  0.000000E+00  0.000000E+00  0.000000E+00  0.000000E+00       Funtag_D
      17  4.042104E+05  3.622609E+05       41949.5       22.7369     0.0122960      0.166570      0.185473      0.242059    0.00182761   -0.00330423    0.00829421       1.02360       Opttag_D
      17       81.3916       1.01171       1.00000  0.000000E+00       1.00000  0.000000E+00      0.942115       102.147       1.00000       36.2898  0.000000E+00      -1.24420  0.000000E+00      -1.24420  0.000000E+00  0.000000E+00       2.48840       82.3604    -0.0687431        1       Scatag_D
      17       1939.87       5721.71  0.000000E+00  0.000000E+00       2619.39  0.000000E+00       2344.12       9589.39  0.000000E+00       12844.6       46.2526       6844.13 -1.000000E+10 -1.000000E+10 -1.000000E+10 -1.000000E+10 -1.000000E+10  0.000000E+00  0.000000E+00  0.000000E+00  0.000000E+00  0.000000E+00  0.000000E+00  0.000000E+00  0.000000E+00  0.000000E+00  0.000000E+00  0.000000E+00  0.000000E+00       Funtag_D
      18  4.041481E+05  3.622465E+05       41901.6       15.0882     0.0128080      0.170680      0.185479      0.242082    0.00186714   -0.00343680    0.00829454       1.02300       Opttag_D
      18       81.3752       1.01032       1.00000  0.000000E+00       1.00000  0.000000E+00      0.942064       102.114       1.00000       36.2691  0.000000E+00      -1.24049  0.000000E+00      -1.24049  0.000000E+00  0.000000E+00       2.48098       82.3604    -0.0687431        1       Scatag_D
      18       1940.03       5714.93  0.000000E+00  0.000000E+00       2619.54  0.000000E+00       2344.25       9546.44  0.000000E+00       12848.8       46.4109       6841.18 -1.000000E+10 -1.000000E+10 -1.000000E+10 -1.000000E+10 -1.000000E+10  0.000000E+00  0.000000E+00  0.000000E+00  0.000000E+00  0.000000E+00  0.000000E+00  0.000000E+00  0.000000E+00  0.000000E+00  0.000000E+00  0.000000E+00  0.000000E+00       Funtag_D
      19  4.040873E+05  3.622272E+05       41860.1       14.4933     0.0134906      0.175437      0.185484      0.242121    0.00191999   -0.00364967    0.00829880       1.02350       Opttag_D
      19       81.3547       1.00854       1.00000  0.000000E+00       1.00000  0.000000E+00      0.942004       102.069       1.00000       36.2411  0.000000E+00      -1.23584  0.000000E+00      -1.23584  0.000000E+00  0.000000E+00       2.47168       82.3604    -0.0687431        1       Scatag_D
      19       1942.02       5720.49  0.000000E+00  0.000000E+00       2619.69  0.000000E+00       2344.16       9495.78  0.000000E+00       12854.2       46.6245       6837.10 -1.000000E+10 -1.000000E+10 -1.000000E+10 -1.000000E+10 -1.000000E+10  0.000000E+00  0.000000E+00  0.000000E+00  0.000000E+00  0.000000E+00  0.000000E+00  0.000000E+00  0.000000E+00  0.000000E+00  0.000000E+00  0.000000E+00  0.000000E+00       Funtag_D
      20  4.040374E+05  3.621978E+05       41839.6       23.5807     0.0146390      0.182870      0.185496      0.242191    0.00200069   -0.00400272    0.00831156       1.02524       Opttag_D
      20       81.3224       1.00571       1.00000  0.000000E+00       1.00000  0.000000E+00      0.941911       101.995       1.00000       36.1950  0.000000E+00      -1.22852  0.000000E+00      -1.22852  0.000000E+00  0.000000E+00       2.45703       82.3604    -0.0687431        1       Scatag_D
      20       1948.00       5739.96  0.000000E+00  0.000000E+00       2620.00  0.000000E+00       2344.06       9446.29  0.000000E+00       12863.0       46.8904       6831.32 -1.000000E+10 -1.000000E+10 -1.000000E+10 -1.000000E+10 -1.000000E+10  0.000000E+00  0.000000E+00  0.000000E+00  0.000000E+00  0.000000E+00  0.000000E+00  0.000000E+00  0.000000E+00  0.000000E+00  0.000000E+00  0.000000E+00  0.000000E+00       Funtag_D
      21  4.039803E+05  3.621803E+05       41800.0       14.9804     0.0153560      0.187195      0.185502      0.242234    0.00204887   -0.00422392    0.00831052       1.02448       Opttag_D
      21       81.3035       1.00402       1.00000  0.000000E+00       1.00000  0.000000E+00      0.941857       101.948       1.00000       36.1671  0.000000E+00      -1.22427  0.000000E+00      -1.22427  0.000000E+00  0.000000E+00       2.44854       82.3604    -0.0687431        1       Scatag_D
      21       1947.51       5731.45  0.000000E+00  0.000000E+00       2620.23  0.000000E+00       2344.08       9413.18  0.000000E+00       12868.7       46.9813       6827.87 -1.000000E+10 -1.000000E+10 -1.000000E+10 -1.000000E+10 -1.000000E+10  0.000000E+00  0.000000E+00  0.000000E+00  0.000000E+00  0.000000E+00  0.000000E+00  0.000000E+00  0.000000E+00  0.000000E+00  0.000000E+00  0.000000E+00  0.000000E+00       Funtag_D
      22  4.039218E+05  3.621551E+05       41766.7       13.7304     0.0163114      0.192046      0.185509      0.242294    0.00211788   -0.00453338    0.00831312       1.02514       Opttag_D
      22       81.2806       1.00182       1.00000  0.000000E+00       1.00000  0.000000E+00      0.941791       101.887       1.00000       36.1298  0.000000E+00      -1.21934  0.000000E+00      -1.21934  0.000000E+00  0.000000E+00       2.43867       82.3604    -0.0687431        1       Scatag_D
      22       1948.72       5738.91  0.000000E+00  0.000000E+00       2620.56  0.000000E+00       2343.82       9368.83  0.000000E+00       12875.4       47.0042       6823.46 -1.000000E+10 -1.000000E+10 -1.000000E+10 -1.000000E+10 -1.000000E+10  0.000000E+00  0.000000E+00  0.000000E+00  0.000000E+00  0.000000E+00  0.000000E+00  0.000000E+00  0.000000E+00  0.000000E+00  0.000000E+00  0.000000E+00  0.000000E+00       Funtag_D
      23  4.038703E+05  3.621231E+05       41747.3       17.3231     0.0175151      0.197314      0.185516      0.242370    0.00220579   -0.00495296    0.00831577       1.02639       Opttag_D
      23       81.2544      0.999144       1.00000  0.000000E+00       1.00000  0.000000E+00      0.941710       101.815       1.00000       36.0848  0.000000E+00      -1.21407  0.000000E+00      -1.21407  0.000000E+00  0.000000E+00       2.42815       82.3604    -0.0687431        1       Scatag_D
      23       1949.97       5752.90  0.000000E+00  0.000000E+00       2621.13  0.000000E+00       2343.43       9330.44  0.000000E+00       12883.9       47.0426       6818.50 -1.000000E+10 -1.000000E+10 -1.000000E+10 -1.000000E+10 -1.000000E+10  0.000000E+00  0.000000E+00  0.000000E+00  0.000000E+00  0.000000E+00  0.000000E+00  0.000000E+00  0.000000E+00  0.000000E+00  0.000000E+00  0.000000E+00  0.000000E+00       Funtag_D
      24  4.038146E+05  3.620827E+05       41731.9       16.0710     0.0191035      0.206726      0.185527      0.242475    0.00231667   -0.00552987    0.00831672       1.02704       Opttag_D
      24       81.2236      0.995782       1.00000  0.000000E+00       1.00000  0.000000E+00      0.941606       101.726       1.00000       36.0288  0.000000E+00      -1.20822  0.000000E+00      -1.20822  0.000000E+00  0.000000E+00       2.41645       82.3604    -0.0687431        1       Scatag_D
      24       1950.41       5760.22  0.000000E+00  0.000000E+00       2621.90  0.000000E+00       2343.05       9301.54  0.000000E+00       12894.6       47.0785       6813.14 -1.000000E+10 -1.000000E+10 -1.000000E+10 -1.000000E+10 -1.000000E+10  0.000000E+00  0.000000E+00  0.000000E+00  0.000000E+00  0.000000E+00  0.000000E+00  0.000000E+00  0.000000E+00  0.000000E+00  0.000000E+00  0.000000E+00  0.000000E+00       Funtag_D
      25  4.037682E+05  3.620561E+05       41712.2       14.4471     0.0199149      0.214603      0.185529      0.242535    0.00238965   -0.00588437    0.00831447       1.02737       Opttag_D
      25       81.2119      0.994061       1.00000  0.000000E+00       1.00000  0.000000E+00      0.941556       101.687       1.00000       36.0009  0.000000E+00      -1.20668  0.000000E+00      -1.20668  0.000000E+00  0.000000E+00       2.41335       82.3604    -0.0687431        1       Scatag_D
      25       1949.36       5763.93  0.000000E+00  0.000000E+00       2622.43  0.000000E+00       2342.72       9278.23  0.000000E+00       12898.9       47.1032       6809.51 -1.000000E+10 -1.000000E+10 -1.000000E+10 -1.000000E+10 -1.000000E+10  0.000000E+00  0.000000E+00  0.000000E+00  0.000000E+00  0.000000E+00  0.000000E+00  0.000000E+00  0.000000E+00  0.000000E+00  0.000000E+00  0.000000E+00  0.000000E+00       Funtag_D
      26  4.037160E+05  3.620188E+05       41697.2       14.4346     0.0209030      0.223272      0.185527      0.242614    0.00249200   -0.00635780    0.00831094       1.02789       Opttag_D
      26       81.2018      0.991989       1.00000  0.000000E+00       1.00000  0.000000E+00      0.941498       101.647       1.00000       35.9684  0.000000E+00      -1.20601  0.000000E+00      -1.20601  0.000000E+00  0.000000E+00       2.41202       82.3604    -0.0687431        1       Scatag_D
      26       1947.71       5769.75  0.000000E+00  0.000000E+00       2623.20  0.000000E+00       2342.49       9257.89  0.000000E+00       12904.3       47.1708       6804.73 -1.000000E+10 -1.000000E+10 -1.000000E+10 -1.000000E+10 -1.000000E+10  0.000000E+00  0.000000E+00  0.000000E+00  0.000000E+00  0.000000E+00  0.000000E+00  0.000000E+00  0.000000E+00  0.000000E+00  0.000000E+00  0.000000E+00  0.000000E+00       Funtag_D
      27  4.036748E+05  3.619852E+05       41689.7       14.8529     0.0218022      0.230313      0.185524      0.242681    0.00258428   -0.00679114    0.00830929       1.02839       Opttag_D
      27       81.1955      0.990232       1.00000  0.000000E+00       1.00000  0.000000E+00      0.941449       101.616       1.00000       35.9408  0.000000E+00      -1.20576  0.000000E+00      -1.20576  0.000000E+00  0.000000E+00       2.41151       82.3604    -0.0687431        1       Scatag_D
      27       1946.93       5775.30  0.000000E+00  0.000000E+00       2623.85  0.000000E+00       2342.95       9243.53  0.000000E+00       12909.3       47.2916       6800.57 -1.000000E+10 -1.000000E+10 -1.000000E+10 -1.000000E+10 -1.000000E+10  0.000000E+00  0.000000E+00  0.000000E+00  0.000000E+00  0.000000E+00  0.000000E+00  0.000000E+00  0.000000E+00  0.000000E+00  0.000000E+00  0.000000E+00  0.000000E+00       Funtag_D
      28  4.036298E+05  3.619505E+05       41679.3       13.1231     0.0227156      0.236488      0.185517      0.242746    0.00267951   -0.00723366    0.00830897       1.02845       Opttag_D
      28       81.1925      0.988592       1.00000  0.000000E+00       1.00000  0.000000E+00      0.941406       101.589       1.00000       35.9145  0.000000E+00      -1.20594  0.000000E+00      -1.20594  0.000000E+00  0.000000E+00       2.41189       82.3604    -0.0687431        1       Scatag_D
      28       1946.78       5775.98  0.000000E+00  0.000000E+00       2624.59  0.000000E+00       2343.98       9229.86  0.000000E+00       12914.2       47.4646       6796.50 -1.000000E+10 -1.000000E+10 -1.000000E+10 -1.000000E+10 -1.000000E+10  0.000000E+00  0.000000E+00  0.000000E+00  0.000000E+00  0.000000E+00  0.000000E+00  0.000000E+00  0.000000E+00  0.000000E+00  0.000000E+00  0.000000E+00  0.000000E+00       Funtag_D
      29  4.035855E+05  3.619087E+05       41676.8       14.5815     0.0239362      0.244249      0.185509      0.242827    0.00279416   -0.00776988    0.00831234       1.02912       Opttag_D
      29       81.1889      0.986597       1.00000  0.000000E+00       1.00000  0.000000E+00      0.941353       101.554       1.00000       35.8813  0.000000E+00      -1.20556  0.000000E+00      -1.20556  0.000000E+00  0.000000E+00       2.41111       82.3604    -0.0687431        1       Scatag_D
      29       1948.36       5783.58  0.000000E+00  0.000000E+00       2625.59  0.000000E+00       2345.78       9211.17  0.000000E+00       12922.4       47.6929       6792.20 -1.000000E+10 -1.000000E+10 -1.000000E+10 -1.000000E+10 -1.000000E+10  0.000000E+00  0.000000E+00  0.000000E+00  0.000000E+00  0.000000E+00  0.000000E+00  0.000000E+00  0.000000E+00  0.000000E+00  0.000000E+00  0.000000E+00  0.000000E+00       Funtag_D
      30  4.035447E+05  3.618744E+05       41670.3       13.3081     0.0250234      0.250718      0.185504      0.242898    0.00288827   -0.00821312    0.00831632       1.02951       Opttag_D
      30       81.1863      0.984987       1.00000  0.000000E+00       1.00000  0.000000E+00      0.941311       101.524       1.00000       35.8531  0.000000E+00      -1.20485  0.000000E+00      -1.20485  0.000000E+00  0.000000E+00       2.40970       82.3604    -0.0687431        1       Scatag_D
      30       1950.23       5787.91  0.000000E+00  0.000000E+00       2626.35  0.000000E+00       2347.34       9191.51  0.000000E+00       12929.8       47.8772       6789.28 -1.000000E+10 -1.000000E+10 -1.000000E+10 -1.000000E+10 -1.000000E+10  0.000000E+00  0.000000E+00  0.000000E+00  0.000000E+00  0.000000E+00  0.000000E+00  0.000000E+00  0.000000E+00  0.000000E+00  0.000000E+00  0.000000E+00  0.000000E+00       Funtag_D
      31  4.035079E+05  3.618395E+05       41668.4       12.7874     0.0261566      0.257015      0.185496      0.242967    0.00298401   -0.00866409    0.00832125       1.02985       Opttag_D
      31       81.1845      0.983412       1.00000  0.000000E+00       1.00000  0.000000E+00      0.941272       101.493       1.00000       35.8248  0.000000E+00      -1.20406  0.000000E+00      -1.20406  0.000000E+00  0.000000E+00       2.40813       82.3604    -0.0687431        1       Scatag_D
      31       1952.54       5791.79  0.000000E+00  0.000000E+00       2627.32  0.000000E+00       2348.80       9175.43  0.000000E+00       12937.8       48.0650       6786.59 -1.000000E+10 -1.000000E+10 -1.000000E+10 -1.000000E+10 -1.000000E+10  0.000000E+00  0.000000E+00  0.000000E+00  0.000000E+00  0.000000E+00  0.000000E+00  0.000000E+00  0.000000E+00  0.000000E+00  0.000000E+00  0.000000E+00  0.000000E+00       Funtag_D
      32  4.034697E+05  3.618005E+05       41669.3       11.9050     0.0273993      0.263387      0.185486      0.243037    0.00309124   -0.00914018    0.00832635       1.03029       Opttag_D
      32       81.1835      0.981756       1.00000  0.000000E+00       1.00000  0.000000E+00      0.941234       101.463       1.00000       35.7946  0.000000E+00      -1.20328  0.000000E+00      -1.20328  0.000000E+00  0.000000E+00       2.40655       82.3604    -0.0687431        1       Scatag_D
      32       1954.94       5796.70  0.000000E+00  0.000000E+00       2628.42  0.000000E+00       2350.04       9159.84  0.000000E+00       12947.2       48.2586       6783.84 -1.000000E+10 -1.000000E+10 -1.000000E+10 -1.000000E+10 -1.000000E+10  0.000000E+00  0.000000E+00  0.000000E+00  0.000000E+00  0.000000E+00  0.000000E+00  0.000000E+00  0.000000E+00  0.000000E+00  0.000000E+00  0.000000E+00  0.000000E+00       Funtag_D
      33  4.034303E+05  3.617485E+05       41681.8       14.6486     0.0291312      0.274892      0.185469      0.243129    0.00323383   -0.00978470    0.00833335       1.03145       Opttag_D
      33       81.1839      0.979600       1.00000  0.000000E+00       1.00000  0.000000E+00      0.941182       101.424       1.00000       35.7552  0.000000E+00      -1.20267  0.000000E+00      -1.20267  0.000000E+00  0.000000E+00       2.40534       82.3604    -0.0687431        1       Scatag_D
      33       1958.22       5809.81  0.000000E+00  0.000000E+00       2629.88  0.000000E+00       2351.91       9142.28  0.000000E+00       12960.3       48.5362       6780.89 -1.000000E+10 -1.000000E+10 -1.000000E+10 -1.000000E+10 -1.000000E+10  0.000000E+00  0.000000E+00  0.000000E+00  0.000000E+00  0.000000E+00  0.000000E+00  0.000000E+00  0.000000E+00  0.000000E+00  0.000000E+00  0.000000E+00  0.000000E+00       Funtag_D
      34  4.033907E+05  3.617079E+05       41682.8       11.8801     0.0305394      0.294552      0.185454      0.243194    0.00334532    -0.0102778    0.00833756       1.03174       Opttag_D
      34       81.1859      0.978009       1.00000  0.000000E+00       1.00000  0.000000E+00      0.941145       101.397       1.00000       35.7246  0.000000E+00      -1.20243  0.000000E+00      -1.20243  0.000000E+00  0.000000E+00       2.40486       82.3604    -0.0687431        1       Scatag_D
      34       1960.20       5813.05  0.000000E+00  0.000000E+00       2631.39  0.000000E+00       2353.26       9127.40  0.000000E+00       12970.4       48.7775       6778.39 -1.000000E+10 -1.000000E+10 -1.000000E+10 -1.000000E+10 -1.000000E+10  0.000000E+00  0.000000E+00  0.000000E+00  0.000000E+00  0.000000E+00  0.000000E+00  0.000000E+00  0.000000E+00  0.000000E+00  0.000000E+00  0.000000E+00  0.000000E+00       Funtag_D
      35  4.033573E+05  3.616786E+05       41678.7       11.3652     0.0315490      0.309064      0.185439      0.243227    0.00342562    -0.0105651    0.00834073       1.03223       Opttag_D
      35       81.1889      0.976997       1.00000  0.000000E+00       1.00000  0.000000E+00      0.941120       101.382       1.00000       35.7023  0.000000E+00      -1.20251  0.000000E+00      -1.20251  0.000000E+00  0.000000E+00       2.40502       82.3604    -0.0687431        1       Scatag_D
      35       1961.69       5818.57  0.000000E+00  0.000000E+00       2632.39  0.000000E+00       2354.21       9109.35  0.000000E+00       12977.4       48.9316       6776.16 -1.000000E+10 -1.000000E+10 -1.000000E+10 -1.000000E+10 -1.000000E+10  0.000000E+00  0.000000E+00  0.000000E+00  0.000000E+00  0.000000E+00  0.000000E+00  0.000000E+00  0.000000E+00  0.000000E+00  0.000000E+00  0.000000E+00  0.000000E+00       Funtag_D
      36  4.033201E+05  3.616305E+05       41689.6       14.1416     0.0334203      0.335936      0.185414      0.243280    0.00355774    -0.0110366    0.00834478       1.03333       Opttag_D
      36       81.1951      0.975367       1.00000  0.000000E+00       1.00000  0.000000E+00      0.941077       101.357       1.00000       35.6629  0.000000E+00      -1.20250  0.000000E+00      -1.20250  0.000000E+00  0.000000E+00       2.40500       82.3604    -0.0687431        1       Scatag_D
      36       1963.60       5830.91  0.000000E+00  0.000000E+00       2634.38  0.000000E+00       2356.18       9092.47  0.000000E+00       12990.1       49.2327       6772.78 -1.000000E+10 -1.000000E+10 -1.000000E+10 -1.000000E+10 -1.000000E+10  0.000000E+00  0.000000E+00  0.000000E+00  0.000000E+00  0.000000E+00  0.000000E+00  0.000000E+00  0.000000E+00  0.000000E+00  0.000000E+00  0.000000E+00  0.000000E+00       Funtag_D
      37  4.032853E+05  3.616009E+05       41684.5       12.3051     0.0346741      0.354226      0.185396      0.243298    0.00363905    -0.0112592    0.00834958       1.03354       Opttag_D
      37       81.2003      0.974479       1.00000  0.000000E+00       1.00000  0.000000E+00      0.941052       101.344       1.00000       35.6365  0.000000E+00      -1.20245  0.000000E+00      -1.20245  0.000000E+00  0.000000E+00       2.40491       82.3604    -0.0687431        1       Scatag_D
      37       1965.86       5833.31  0.000000E+00  0.000000E+00       2635.78  0.000000E+00       2357.46       9074.28  0.000000E+00       12998.3       49.3967       6770.10 -1.000000E+10 -1.000000E+10 -1.000000E+10 -1.000000E+10 -1.000000E+10  0.000000E+00  0.000000E+00  0.000000E+00  0.000000E+00  0.000000E+00  0.000000E+00  0.000000E+00  0.000000E+00  0.000000E+00  0.000000E+00  0.000000E+00  0.000000E+00       Funtag_D
      38  4.032539E+05  3.615832E+05       41670.7       10.8887     0.0354039      0.365265      0.185383      0.243297    0.00368743    -0.0113268    0.00835041       1.03372       Opttag_D
      38       81.2040      0.974052       1.00000  0.000000E+00       1.00000  0.000000E+00      0.941040       101.339       1.00000       35.6205  0.000000E+00      -1.20241  0.000000E+00      -1.20241  0.000000E+00  0.000000E+00       2.40482       82.3604    -0.0687431        1       Scatag_D
      38       1966.25       5835.35  0.000000E+00  0.000000E+00       2636.59  0.000000E+00       2358.10       9054.04  0.000000E+00       13002.8       49.4890       6768.08 -1.000000E+10 -1.000000E+10 -1.000000E+10 -1.000000E+10 -1.000000E+10  0.000000E+00  0.000000E+00  0.000000E+00  0.000000E+00  0.000000E+00  0.000000E+00  0.000000E+00  0.000000E+00  0.000000E+00  0.000000E+00  0.000000E+00  0.000000E+00       Funtag_D
      39  4.032122E+05  3.615407E+05       41671.5       13.4061     0.0373185      0.393578      0.185357      0.243307    0.00380411    -0.0115701    0.00835465       1.03461       Opttag_D
      39       81.2129      0.972892       1.00000  0.000000E+00       1.00000  0.000000E+00      0.941009       101.321       1.00000       35.5831  0.000000E+00      -1.20229  0.000000E+00      -1.20229  0.000000E+00  0.000000E+00       2.40458       82.3604    -0.0687431        1       Scatag_D
      39       1968.25       5845.40  0.000000E+00  0.000000E+00       2638.82  0.000000E+00       2359.95       9029.77  0.000000E+00       13015.3       49.7766       6764.27 -1.000000E+10 -1.000000E+10 -1.000000E+10 -1.000000E+10 -1.000000E+10  0.000000E+00  0.000000E+00  0.000000E+00  0.000000E+00  0.000000E+00  0.000000E+00  0.000000E+00  0.000000E+00  0.000000E+00  0.000000E+00  0.000000E+00  0.000000E+00       Funtag_D
      40  4.031881E+05  3.615036E+05       41684.5       16.4196     0.0392276      0.421597      0.185333      0.243318    0.00390594    -0.0118202    0.00836264       1.03537       Opttag_D
      40       81.2223      0.971851       1.00000  0.000000E+00       1.00000  0.000000E+00      0.940983       101.303       1.00000       35.5496  0.000000E+00      -1.20219  0.000000E+00      -1.20219  0.000000E+00  0.000000E+00       2.40437       82.3604    -0.0687431        1       Scatag_D
      40       1972.01       5853.99  0.000000E+00  0.000000E+00       2641.28  0.000000E+00       2361.90       9016.63  0.000000E+00       13027.5       50.0672       6761.04 -1.000000E+10 -1.000000E+10 -1.000000E+10 -1.000000E+10 -1.000000E+10  0.000000E+00  0.000000E+00  0.000000E+00  0.000000E+00  0.000000E+00  0.000000E+00  0.000000E+00  0.000000E+00  0.000000E+00  0.000000E+00  0.000000E+00  0.000000E+00       Funtag_D
      41  4.031601E+05  3.614958E+05       41664.4       10.3454     0.0395256      0.426071      0.185330      0.243320    0.00392749    -0.0118686    0.00836010       1.03509       Opttag_D
      41       81.2238      0.971612       1.00000  0.000000E+00       1.00000  0.000000E+00      0.940981       101.298       1.00000       35.5450  0.000000E+00      -1.20229  0.000000E+00      -1.20229  0.000000E+00  0.000000E+00       2.40458       82.3604    -0.0687431        1       Scatag_D
      41       1970.81       5850.88  0.000000E+00  0.000000E+00       2641.60  0.000000E+00       2362.19       8999.33  0.000000E+00       13029.4       50.1169       6760.08 -1.000000E+10 -1.000000E+10 -1.000000E+10 -1.000000E+10 -1.000000E+10  0.000000E+00  0.000000E+00  0.000000E+00  0.000000E+00  0.000000E+00  0.000000E+00  0.000000E+00  0.000000E+00  0.000000E+00  0.000000E+00  0.000000E+00  0.000000E+00       Funtag_D
      42  4.031244E+05  3.614701E+05       41654.3       9.18213     0.0407446      0.443976      0.185317      0.243334    0.00399803    -0.0120671    0.00836149       1.03558       Opttag_D
      42       81.2300      0.970823       1.00000  0.000000E+00       1.00000  0.000000E+00      0.940968       101.283       1.00000       35.5269  0.000000E+00      -1.20248  0.000000E+00      -1.20248  0.000000E+00  0.000000E+00       2.40496       82.3604    -0.0687431        1       Scatag_D
      42       1971.47       5856.37  0.000000E+00  0.000000E+00       2643.13  0.000000E+00       2363.63       8975.04  0.000000E+00       13036.8       50.3041       6757.50 -1.000000E+10 -1.000000E+10 -1.000000E+10 -1.000000E+10 -1.000000E+10  0.000000E+00  0.000000E+00  0.000000E+00  0.000000E+00  0.000000E+00  0.000000E+00  0.000000E+00  0.000000E+00  0.000000E+00  0.000000E+00  0.000000E+00  0.000000E+00       Funtag_D
      43  4.030937E+05  3.614397E+05       41654.0       11.1297     0.0424201      0.468411      0.185300      0.243348    0.00408137    -0.0122911    0.00836656       1.03616       Opttag_D
      43       81.2391      0.969913       1.00000  0.000000E+00       1.00000  0.000000E+00      0.940953       101.262       1.00000       35.5033  0.000000E+00      -1.20265  0.000000E+00      -1.20265  0.000000E+00  0.000000E+00       2.40529       82.3604    -0.0687431        1       Scatag_D
      43       1973.86       5862.96  0.000000E+00  0.000000E+00       2645.29  0.000000E+00       2365.90       8954.25  0.000000E+00       13046.8       50.5110       6754.43 -1.000000E+10 -1.000000E+10 -1.000000E+10 -1.000000E+10 -1.000000E+10  0.000000E+00  0.000000E+00  0.000000E+00  0.000000E+00  0.000000E+00  0.000000E+00  0.000000E+00  0.000000E+00  0.000000E+00  0.000000E+00  0.000000E+00  0.000000E+00       Funtag_D
      44  4.030566E+05  3.613892E+05       41667.4       14.9391     0.0456851      0.515453      0.185274      0.243383    0.00422000    -0.0127483    0.00837510       1.03753       Opttag_D
      44       81.2573      0.968343       1.00000  0.000000E+00       1.00000  0.000000E+00      0.940928       101.218       1.00000       35.4639  0.000000E+00      -1.20290  0.000000E+00      -1.20290  0.000000E+00  0.000000E+00       2.40579       82.3604    -0.0687431        1       Scatag_D
      44       1977.89       5878.45  0.000000E+00  0.000000E+00       2649.58  0.000000E+00       2370.95       8923.91  0.000000E+00       13066.1       50.8586       6749.60 -1.000000E+10 -1.000000E+10 -1.000000E+10 -1.000000E+10 -1.000000E+10  0.000000E+00  0.000000E+00  0.000000E+00  0.000000E+00  0.000000E+00  0.000000E+00  0.000000E+00  0.000000E+00  0.000000E+00  0.000000E+00  0.000000E+00  0.000000E+00       Funtag_D
      45  4.030216E+05  3.613735E+05       41648.1       10.6176     0.0467391      0.530571      0.185266      0.243386    0.00426309    -0.0128458    0.00837586       1.03756       Opttag_D
      45       81.2639      0.967873       1.00000  0.000000E+00       1.00000  0.000000E+00      0.940926       101.201       1.00000       35.4532  0.000000E+00      -1.20313  0.000000E+00      -1.20313  0.000000E+00  0.000000E+00       2.40626       82.3604    -0.0687431        1       Scatag_D
      45       1978.25       5878.84  0.000000E+00  0.000000E+00       2651.06  0.000000E+00       2372.78       8897.96  0.000000E+00       13070.8       50.8805       6747.52 -1.000000E+10 -1.000000E+10 -1.000000E+10 -1.000000E+10 -1.000000E+10  0.000000E+00  0.000000E+00  0.000000E+00  0.000000E+00  0.000000E+00  0.000000E+00  0.000000E+00  0.000000E+00  0.000000E+00  0.000000E+00  0.000000E+00  0.000000E+00       Funtag_D
      46  4.029885E+05  3.613603E+05       41628.2       10.2520     0.0475091      0.541730      0.185260      0.243381    0.00429938    -0.0129021    0.00837622       1.03773       Opttag_D
      46       81.2692      0.967513       1.00000  0.000000E+00       1.00000  0.000000E+00      0.940932       101.185       1.00000       35.4467  0.000000E+00      -1.20352  0.000000E+00      -1.20352  0.000000E+00  0.000000E+00       2.40704       82.3604    -0.0687431        1       Scatag_D
      46       1978.42       5880.71  0.000000E+00  0.000000E+00       2652.16  0.000000E+00       2374.24       8874.46  0.000000E+00       13072.3       50.8290       6745.05 -1.000000E+10 -1.000000E+10 -1.000000E+10 -1.000000E+10 -1.000000E+10  0.000000E+00  0.000000E+00  0.000000E+00  0.000000E+00  0.000000E+00  0.000000E+00  0.000000E+00  0.000000E+00  0.000000E+00  0.000000E+00  0.000000E+00  0.000000E+00       Funtag_D
      47  4.029533E+05  3.613380E+05       41615.4       12.3836     0.0488828      0.565017      0.185251      0.243379    0.00436066    -0.0130077    0.00837646       1.03803       Opttag_D
      47       81.2788      0.966890       1.00000  0.000000E+00       1.00000  0.000000E+00      0.940941       101.158       1.00000       35.4369  0.000000E+00      -1.20451  0.000000E+00      -1.20451  0.000000E+00  0.000000E+00       2.40902       82.3604    -0.0687431        1       Scatag_D
      47       1978.53       5884.15  0.000000E+00  0.000000E+00       2654.08  0.000000E+00       2376.73       8854.62  0.000000E+00       13075.2       50.8203       6741.21 -1.000000E+10 -1.000000E+10 -1.000000E+10 -1.000000E+10 -1.000000E+10  0.000000E+00  0.000000E+00  0.000000E+00  0.000000E+00  0.000000E+00  0.000000E+00  0.000000E+00  0.000000E+00  0.000000E+00  0.000000E+00  0.000000E+00  0.000000E+00       Funtag_D
      48  4.029201E+05  3.613101E+05       41610.0       15.5796     0.0508478      0.601024      0.185244      0.243379    0.00443709    -0.0131907    0.00837538       1.03867       Opttag_D
      48       81.2928      0.966096       1.00000  0.000000E+00       1.00000  0.000000E+00      0.940956       101.121       1.00000       35.4260  0.000000E+00      -1.20591  0.000000E+00      -1.20591  0.000000E+00  0.000000E+00       2.41182       82.3604    -0.0687431        1       Scatag_D
      48       1978.02       5891.35  0.000000E+00  0.000000E+00       2656.88  0.000000E+00       2380.12       8836.43  0.000000E+00       13079.4       50.8910       6736.83 -1.000000E+10 -1.000000E+10 -1.000000E+10 -1.000000E+10 -1.000000E+10  0.000000E+00  0.000000E+00  0.000000E+00  0.000000E+00  0.000000E+00  0.000000E+00  0.000000E+00  0.000000E+00  0.000000E+00  0.000000E+00  0.000000E+00  0.000000E+00       Funtag_D
      49  4.028868E+05  3.613004E+05       41586.5       11.2080     0.0515081      0.613618      0.185241      0.243374    0.00446382    -0.0132417    0.00837359       1.03869       Opttag_D
      49       81.2978      0.965837       1.00000  0.000000E+00       1.00000  0.000000E+00      0.940963       101.110       1.00000       35.4232  0.000000E+00      -1.20672  0.000000E+00      -1.20672  0.000000E+00  0.000000E+00       2.41343       82.3604    -0.0687431        1       Scatag_D
      49       1977.18       5891.56  0.000000E+00  0.000000E+00       2657.80  0.000000E+00       2380.89       8813.21  0.000000E+00       13080.0       50.9755       6734.86 -1.000000E+10 -1.000000E+10 -1.000000E+10 -1.000000E+10 -1.000000E+10  0.000000E+00  0.000000E+00  0.000000E+00  0.000000E+00  0.000000E+00  0.000000E+00  0.000000E+00  0.000000E+00  0.000000E+00  0.000000E+00  0.000000E+00  0.000000E+00       Funtag_D
      50  4.028488E+05  3.612817E+05       41567.0       9.87075     0.0527355      0.636883      0.185236      0.243365    0.00451494    -0.0133569    0.00837273       1.03900       Opttag_D
      50       81.3073      0.965352       1.00000  0.000000E+00       1.00000  0.000000E+00      0.940976       101.089       1.00000       35.4183  0.000000E+00      -1.20817  0.000000E+00      -1.20817  0.000000E+00  0.000000E+00       2.41633       82.3604    -0.0687431        1       Scatag_D
      50       1976.77       5895.13  0.000000E+00  0.000000E+00       2659.56  0.000000E+00       2382.04       8789.81  0.000000E+00       13081.2       51.1568       6731.37 -1.000000E+10 -1.000000E+10 -1.000000E+10 -1.000000E+10 -1.000000E+10  0.000000E+00  0.000000E+00  0.000000E+00  0.000000E+00  0.000000E+00  0.000000E+00  0.000000E+00  0.000000E+00  0.000000E+00  0.000000E+00  0.000000E+00  0.000000E+00       Funtag_D
      51  4.028235E+05  3.612630E+05       41560.5       11.5896     0.0541563      0.663152      0.185230      0.243355    0.00456627    -0.0134626    0.00837127       1.03932       Opttag_D
      51       81.3186      0.964917       1.00000  0.000000E+00       1.00000  0.000000E+00      0.940992       101.066       1.00000       35.4125  0.000000E+00      -1.20936  0.000000E+00      -1.20936  0.000000E+00  0.000000E+00       2.41873       82.3604    -0.0687431        1       Scatag_D
      51       1976.09       5898.76  0.000000E+00  0.000000E+00       2661.62  0.000000E+00       2383.51       8778.18  0.000000E+00       13083.2       51.2526       6727.91 -1.000000E+10 -1.000000E+10 -1.000000E+10 -1.000000E+10 -1.000000E+10  0.000000E+00  0.000000E+00  0.000000E+00  0.000000E+00  0.000000E+00  0.000000E+00  0.000000E+00  0.000000E+00  0.000000E+00  0.000000E+00  0.000000E+00  0.000000E+00       Funtag_D
      52  4.027936E+05  3.612460E+05       41547.6       9.00925     0.0554743      0.687232      0.185223      0.243351    0.00461292    -0.0135845    0.00837091       1.03953       Opttag_D
      52       81.3295      0.964567       1.00000  0.000000E+00       1.00000  0.000000E+00      0.941009       101.045       1.00000       35.4073  0.000000E+00      -1.21037  0.000000E+00      -1.21037  0.000000E+00  0.000000E+00       2.42073       82.3604    -0.0687431        1       Scatag_D
      52       1975.92       5901.17  0.000000E+00  0.000000E+00       2663.50  0.000000E+00       2384.50       8761.99  0.000000E+00       13084.7       51.3058       6724.49 -1.000000E+10 -1.000000E+10 -1.000000E+10 -1.000000E+10 -1.000000E+10  0.000000E+00  0.000000E+00  0.000000E+00  0.000000E+00  0.000000E+00  0.000000E+00  0.000000E+00  0.000000E+00  0.000000E+00  0.000000E+00  0.000000E+00  0.000000E+00       Funtag_D
      53  4.027653E+05  3.612281E+05       41537.1       10.1740     0.0566982      0.709472      0.185215      0.243343    0.00466211    -0.0136899    0.00837068       1.03969       Opttag_D
      53       81.3402      0.964261       1.00000  0.000000E+00       1.00000  0.000000E+00      0.941029       101.024       1.00000       35.4020  0.000000E+00      -1.21099  0.000000E+00      -1.21099  0.000000E+00  0.000000E+00       2.42197       82.3604    -0.0687431        1       Scatag_D
      53       1975.81       5902.91  0.000000E+00  0.000000E+00       2665.17  0.000000E+00       2385.03       8750.08  0.000000E+00       13085.9       51.2582       6721.02 -1.000000E+10 -1.000000E+10 -1.000000E+10 -1.000000E+10 -1.000000E+10  0.000000E+00  0.000000E+00  0.000000E+00  0.000000E+00  0.000000E+00  0.000000E+00  0.000000E+00  0.000000E+00  0.000000E+00  0.000000E+00  0.000000E+00  0.000000E+00       Funtag_D
      54  4.027357E+05  3.612089E+05       41526.8       12.5862     0.0582361      0.737720      0.185203      0.243334    0.00471476    -0.0137918    0.00836889       1.03986       Opttag_D
      54       81.3544      0.964028       1.00000  0.000000E+00       1.00000  0.000000E+00      0.941054       100.997       1.00000       35.3956  0.000000E+00      -1.21180  0.000000E+00      -1.21180  0.000000E+00  0.000000E+00       2.42359       82.3604    -0.0687431        1       Scatag_D
      54       1974.96       5904.86  0.000000E+00  0.000000E+00       2667.25  0.000000E+00       2385.44       8739.68  0.000000E+00       13086.8       51.1567       6716.63 -1.000000E+10 -1.000000E+10 -1.000000E+10 -1.000000E+10 -1.000000E+10  0.000000E+00  0.000000E+00  0.000000E+00  0.000000E+00  0.000000E+00  0.000000E+00  0.000000E+00  0.000000E+00  0.000000E+00  0.000000E+00  0.000000E+00  0.000000E+00       Funtag_D
      55  4.027070E+05  3.611968E+05       41510.2       10.9896     0.0590662      0.753524      0.185195      0.243327    0.00474799    -0.0138433    0.00836750       1.03988       Opttag_D
      55       81.3627      0.963888       1.00000  0.000000E+00       1.00000  0.000000E+00      0.941071       100.982       1.00000       35.3927  0.000000E+00      -1.21240  0.000000E+00      -1.21240  0.000000E+00  0.000000E+00       2.42481       82.3604    -0.0687431        1       Scatag_D
      55       1974.30       5905.11  0.000000E+00  0.000000E+00       2668.30  0.000000E+00       2385.35       8725.48  0.000000E+00       13086.6       51.0962       6714.00 -1.000000E+10 -1.000000E+10 -1.000000E+10 -1.000000E+10 -1.000000E+10  0.000000E+00  0.000000E+00  0.000000E+00  0.000000E+00  0.000000E+00  0.000000E+00  0.000000E+00  0.000000E+00  0.000000E+00  0.000000E+00  0.000000E+00  0.000000E+00       Funtag_D
      56  4.026669E+05  3.611761E+05       41490.9       10.9654     0.0604488      0.780357      0.185181      0.243315    0.00480491    -0.0139100    0.00836504       1.04002       Opttag_D
      56       81.3770      0.963672       1.00000  0.000000E+00       1.00000  0.000000E+00      0.941100       100.953       1.00000       35.3886  0.000000E+00      -1.21384  0.000000E+00      -1.21384  0.000000E+00  0.000000E+00       2.42767       82.3604    -0.0687431        1       Scatag_D
      56       1973.14       5906.69  0.000000E+00  0.000000E+00       2670.00  0.000000E+00       2385.05       8709.83  0.000000E+00       13085.5       51.0243       6709.66 -1.000000E+10 -1.000000E+10 -1.000000E+10 -1.000000E+10 -1.000000E+10  0.000000E+00  0.000000E+00  0.000000E+00  0.000000E+00  0.000000E+00  0.000000E+00  0.000000E+00  0.000000E+00  0.000000E+00  0.000000E+00  0.000000E+00  0.000000E+00       Funtag_D
      57  4.026393E+05  3.611594E+05       41479.9       14.6422     0.0621160      0.811914      0.185168      0.243301    0.00485075    -0.0139924    0.00836232       1.04040       Opttag_D
      57       81.3942      0.963561       1.00000  0.000000E+00       1.00000  0.000000E+00      0.941131       100.922       1.00000       35.3855  0.000000E+00      -1.21595  0.000000E+00      -1.21595  0.000000E+00  0.000000E+00       2.43190       82.3604    -0.0687431        1       Scatag_D
      57       1971.86       5910.97  0.000000E+00  0.000000E+00       2672.18  0.000000E+00       2385.27       8698.09  0.000000E+00       13085.1       51.0561       6705.36 -1.000000E+10 -1.000000E+10 -1.000000E+10 -1.000000E+10 -1.000000E+10  0.000000E+00  0.000000E+00  0.000000E+00  0.000000E+00  0.000000E+00  0.000000E+00  0.000000E+00  0.000000E+00  0.000000E+00  0.000000E+00  0.000000E+00  0.000000E+00       Funtag_D
      58  4.026103E+05  3.611502E+05       41460.1       10.0620     0.0628379      0.825566      0.185163      0.243294    0.00487585    -0.0140341    0.00836060       1.04042       Opttag_D
      58       81.4018      0.963462       1.00000  0.000000E+00       1.00000  0.000000E+00      0.941144       100.907       1.00000       35.3843  0.000000E+00      -1.21721  0.000000E+00      -1.21721  0.000000E+00  0.000000E+00       2.43442       82.3604    -0.0687431        1       Scatag_D
      58       1971.05       5911.20  0.000000E+00  0.000000E+00       2673.12  0.000000E+00       2385.31       8680.85  0.000000E+00       13084.5       51.0865       6703.04 -1.000000E+10 -1.000000E+10 -1.000000E+10 -1.000000E+10 -1.000000E+10  0.000000E+00  0.000000E+00  0.000000E+00  0.000000E+00  0.000000E+00  0.000000E+00  0.000000E+00  0.000000E+00  0.000000E+00  0.000000E+00  0.000000E+00  0.000000E+00       Funtag_D
      59  4.025828E+05  3.611376E+05       41445.2       8.97795     0.0638456      0.843888      0.185155      0.243283    0.00491051    -0.0140948    0.00835990       1.04056       Opttag_D
      59       81.4124      0.963319       1.00000  0.000000E+00       1.00000  0.000000E+00      0.941160       100.886       1.00000       35.3820  0.000000E+00      -1.21896  0.000000E+00      -1.21896  0.000000E+00  0.000000E+00       2.43791       82.3604    -0.0687431        1       Scatag_D
      59       1970.72       5912.82  0.000000E+00  0.000000E+00       2674.46  0.000000E+00       2385.64       8666.68  0.000000E+00       13084.0       51.1213       6699.74 -1.000000E+10 -1.000000E+10 -1.000000E+10 -1.000000E+10 -1.000000E+10  0.000000E+00  0.000000E+00  0.000000E+00  0.000000E+00  0.000000E+00  0.000000E+00  0.000000E+00  0.000000E+00  0.000000E+00  0.000000E+00  0.000000E+00  0.000000E+00       Funtag_D
      60  4.025609E+05  3.611258E+05       41435.1       9.80579     0.0649222      0.862708      0.185144      0.243273    0.00494303    -0.0141580    0.00835888       1.04078       Opttag_D
      60       81.4238      0.963227       1.00000  0.000000E+00       1.00000  0.000000E+00      0.941177       100.865       1.00000       35.3793  0.000000E+00      -1.22051  0.000000E+00      -1.22051  0.000000E+00  0.000000E+00       2.44102       82.3604    -0.0687431        1       Scatag_D
      60       1970.24       5915.38  0.000000E+00  0.000000E+00       2675.89  0.000000E+00       2386.22       8655.74  0.000000E+00       13084.0       51.0937       6696.53 -1.000000E+10 -1.000000E+10 -1.000000E+10 -1.000000E+10 -1.000000E+10  0.000000E+00  0.000000E+00  0.000000E+00  0.000000E+00  0.000000E+00  0.000000E+00  0.000000E+00  0.000000E+00  0.000000E+00  0.000000E+00  0.000000E+00  0.000000E+00       Funtag_D
      61  4.025284E+05  3.611039E+05       41424.4       12.1630     0.0673318      0.903530      0.185124      0.243262    0.00500289    -0.0143059    0.00836096       1.04121       Opttag_D
      61       81.4493      0.963129       1.00000  0.000000E+00       1.00000  0.000000E+00      0.941211       100.816       1.00000       35.3752  0.000000E+00      -1.22361  0.000000E+00      -1.22361  0.000000E+00  0.000000E+00       2.44723       82.3604    -0.0687431        1       Scatag_D
      61       1971.22       5920.20  0.000000E+00  0.000000E+00       2679.05  0.000000E+00       2387.85       8639.88  0.000000E+00       13085.1       50.9592       6690.23 -1.000000E+10 -1.000000E+10 -1.000000E+10 -1.000000E+10 -1.000000E+10  0.000000E+00  0.000000E+00  0.000000E+00  0.000000E+00  0.000000E+00  0.000000E+00  0.000000E+00  0.000000E+00  0.000000E+00  0.000000E+00  0.000000E+00  0.000000E+00       Funtag_D
      62  4.025001E+05  3.610917E+05       41408.4       10.8247     0.0687617      0.926840      0.185114      0.243259    0.00503633    -0.0143786    0.00836187       1.04140       Opttag_D
      62       81.4649      0.963108       1.00000  0.000000E+00       1.00000  0.000000E+00      0.941228       100.786       1.00000       35.3727  0.000000E+00      -1.22535  0.000000E+00      -1.22535  0.000000E+00  0.000000E+00       2.45070       82.3604    -0.0687431        1       Scatag_D
      62       1971.65       5922.38  0.000000E+00  0.000000E+00       2680.82  0.000000E+00       2389.05       8620.65  0.000000E+00       13085.9       50.8385       6687.08 -1.000000E+10 -1.000000E+10 -1.000000E+10 -1.000000E+10 -1.000000E+10  0.000000E+00  0.000000E+00  0.000000E+00  0.000000E+00  0.000000E+00  0.000000E+00  0.000000E+00  0.000000E+00  0.000000E+00  0.000000E+00  0.000000E+00  0.000000E+00       Funtag_D
      63  4.024760E+05  3.610858E+05       41390.2       8.59159     0.0693957      0.937003      0.185109      0.243264    0.00505270    -0.0144242    0.00836146       1.04144       Opttag_D
      63       81.4723      0.963099       1.00000  0.000000E+00       1.00000  0.000000E+00      0.941235       100.772       1.00000       35.3713  0.000000E+00      -1.22622  0.000000E+00      -1.22622  0.000000E+00  0.000000E+00       2.45243       82.3604    -0.0687431        1       Scatag_D
      63       1971.46       5922.82  0.000000E+00  0.000000E+00       2681.55  0.000000E+00       2389.49       8602.18  0.000000E+00       13086.1       50.7720       6685.78 -1.000000E+10 -1.000000E+10 -1.000000E+10 -1.000000E+10 -1.000000E+10  0.000000E+00  0.000000E+00  0.000000E+00  0.000000E+00  0.000000E+00  0.000000E+00  0.000000E+00  0.000000E+00  0.000000E+00  0.000000E+00  0.000000E+00  0.000000E+00       Funtag_D
      64  4.024494E+05  3.610755E+05       41373.9       9.12256     0.0704103      0.953371      0.185100      0.243273    0.00508105    -0.0144848    0.00836262       1.04146       Opttag_D
      64       81.4842      0.963046       1.00000  0.000000E+00       1.00000  0.000000E+00      0.941245       100.748       1.00000       35.3694  0.000000E+00      -1.22779  0.000000E+00      -1.22779  0.000000E+00  0.000000E+00       2.45558       82.3604    -0.0687431        1       Scatag_D
      64       1972.00       5923.04  0.000000E+00  0.000000E+00       2682.70  0.000000E+00       2389.97       8585.41  0.000000E+00       13086.2       50.7125       6683.87 -1.000000E+10 -1.000000E+10 -1.000000E+10 -1.000000E+10 -1.000000E+10  0.000000E+00  0.000000E+00  0.000000E+00  0.000000E+00  0.000000E+00  0.000000E+00  0.000000E+00  0.000000E+00  0.000000E+00  0.000000E+00  0.000000E+00  0.000000E+00       Funtag_D
      65  4.024299E+05  3.610612E+05       41368.8       14.3557     0.0719254      0.977351      0.185089      0.243288    0.00512028    -0.0145898    0.00836710       1.04165       Opttag_D
      65       81.5021      0.962976       1.00000  0.000000E+00       1.00000  0.000000E+00      0.941260       100.712       1.00000       35.3669  0.000000E+00      -1.23039  0.000000E+00      -1.23039  0.000000E+00  0.000000E+00       2.46078       82.3604    -0.0687431        1       Scatag_D
      65       1974.12       5925.26  0.000000E+00  0.000000E+00       2684.35  0.000000E+00       2390.56       8575.50  0.000000E+00       13086.6       50.7278       6681.62 -1.000000E+10 -1.000000E+10 -1.000000E+10 -1.000000E+10 -1.000000E+10  0.000000E+00  0.000000E+00  0.000000E+00  0.000000E+00  0.000000E+00  0.000000E+00  0.000000E+00  0.000000E+00  0.000000E+00  0.000000E+00  0.000000E+00  0.000000E+00       Funtag_D
      66  4.024026E+05  3.610540E+05       41348.7       9.64160     0.0726554      0.988753      0.185082      0.243289    0.00514000    -0.0146215    0.00836797       1.04166       Opttag_D
      66       81.5108      0.962923       1.00000  0.000000E+00       1.00000  0.000000E+00      0.941268       100.694       1.00000       35.3658  0.000000E+00      -1.23182  0.000000E+00      -1.23182  0.000000E+00  0.000000E+00       2.46365       82.3604    -0.0687431        1       Scatag_D
      66       1974.53       5925.36  0.000000E+00  0.000000E+00       2685.18  0.000000E+00       2390.64       8554.90  0.000000E+00       13086.8       50.7736       6680.47 -1.000000E+10 -1.000000E+10 -1.000000E+10 -1.000000E+10 -1.000000E+10  0.000000E+00  0.000000E+00  0.000000E+00  0.000000E+00  0.000000E+00  0.000000E+00  0.000000E+00  0.000000E+00  0.000000E+00  0.000000E+00  0.000000E+00  0.000000E+00       Funtag_D
      67  4.023679E+05  3.610400E+05       41327.8       8.11799     0.0742440       1.01282      0.185071      0.243296    0.00517832    -0.0146787    0.00837136       1.04190       Opttag_D
      67       81.5297      0.962855       1.00000  0.000000E+00       1.00000  0.000000E+00      0.941285       100.656       1.00000       35.3631  0.000000E+00      -1.23487  0.000000E+00      -1.23487  0.000000E+00  0.000000E+00       2.46973       82.3604    -0.0687431        1       Scatag_D
      67       1976.13       5928.08  0.000000E+00  0.000000E+00       2686.96  0.000000E+00       2390.75       8529.37  0.000000E+00       13087.6       50.8915       6678.02 -1.000000E+10 -1.000000E+10 -1.000000E+10 -1.000000E+10 -1.000000E+10  0.000000E+00  0.000000E+00  0.000000E+00  0.000000E+00  0.000000E+00  0.000000E+00  0.000000E+00  0.000000E+00  0.000000E+00  0.000000E+00  0.000000E+00  0.000000E+00       Funtag_D
      68  4.023441E+05  3.610285E+05       41315.6       9.33701     0.0756785       1.03312      0.185062      0.243300    0.00521003    -0.0147115    0.00837386       1.04210       Opttag_D
      68       81.5471      0.962857       1.00000  0.000000E+00       1.00000  0.000000E+00      0.941301       100.622       1.00000       35.3599  0.000000E+00      -1.23740  0.000000E+00      -1.23740  0.000000E+00  0.000000E+00       2.47480       82.3604    -0.0687431        1       Scatag_D
      68       1977.31       5930.35  0.000000E+00  0.000000E+00       2688.53  0.000000E+00       2390.90       8513.09  0.000000E+00       13088.8       50.9155       6675.69 -1.000000E+10 -1.000000E+10 -1.000000E+10 -1.000000E+10 -1.000000E+10  0.000000E+00  0.000000E+00  0.000000E+00  0.000000E+00  0.000000E+00  0.000000E+00  0.000000E+00  0.000000E+00  0.000000E+00  0.000000E+00  0.000000E+00  0.000000E+00       Funtag_D
      69  4.023146E+05  3.610092E+05       41305.4       12.2498     0.0786193       1.07281      0.185042      0.243302    0.00526288    -0.0147501    0.00837896       1.04268       Opttag_D
      69       81.5828      0.963005       1.00000  0.000000E+00       1.00000  0.000000E+00      0.941334       100.555       1.00000       35.3545  0.000000E+00      -1.24205  0.000000E+00      -1.24205  0.000000E+00  0.000000E+00       2.48409       82.3604    -0.0687431        1       Scatag_D
      69       1979.72       5936.98  0.000000E+00  0.000000E+00       2691.70  0.000000E+00       2391.17       8490.77  0.000000E+00       13092.7       50.9165       6671.43 -1.000000E+10 -1.000000E+10 -1.000000E+10 -1.000000E+10 -1.000000E+10  0.000000E+00  0.000000E+00  0.000000E+00  0.000000E+00  0.000000E+00  0.000000E+00  0.000000E+00  0.000000E+00  0.000000E+00  0.000000E+00  0.000000E+00  0.000000E+00       Funtag_D
      70  4.022896E+05  3.610022E+05       41287.4       9.24184     0.0792947       1.08069      0.185033      0.243292    0.00528219    -0.0147245    0.00837824       1.04277       Opttag_D
      70       81.5918      0.963044       1.00000  0.000000E+00       1.00000  0.000000E+00      0.941342       100.541       1.00000       35.3503  0.000000E+00      -1.24309  0.000000E+00      -1.24309  0.000000E+00  0.000000E+00       2.48618       82.3604    -0.0687431        1       Scatag_D
      70       1979.37       5937.96  0.000000E+00  0.000000E+00       2692.35  0.000000E+00       2390.77       8473.12  0.000000E+00       13093.4       50.8531       6669.54 -1.000000E+10 -1.000000E+10 -1.000000E+10 -1.000000E+10 -1.000000E+10  0.000000E+00  0.000000E+00  0.000000E+00  0.000000E+00  0.000000E+00  0.000000E+00  0.000000E+00  0.000000E+00  0.000000E+00  0.000000E+00  0.000000E+00  0.000000E+00       Funtag_D
      71  4.022677E+05  3.609927E+05       41275.0       8.66281     0.0796639       1.08410      0.185024      0.243282    0.00530821    -0.0146985    0.00837601       1.04283       Opttag_D
      71       81.5973      0.963001       1.00000  0.000000E+00       1.00000  0.000000E+00      0.941348       100.534       1.00000       35.3449  0.000000E+00      -1.24361  0.000000E+00      -1.24361  0.000000E+00  0.000000E+00       2.48723       82.3604    -0.0687431        1       Scatag_D
      71       1978.32       5938.69  0.000000E+00  0.000000E+00       2692.66  0.000000E+00       2390.06       8463.31  0.000000E+00       13093.6       50.7726       6667.60 -1.000000E+10 -1.000000E+10 -1.000000E+10 -1.000000E+10 -1.000000E+10  0.000000E+00  0.000000E+00  0.000000E+00  0.000000E+00  0.000000E+00  0.000000E+00  0.000000E+00  0.000000E+00  0.000000E+00  0.000000E+00  0.000000E+00  0.000000E+00       Funtag_D
      72  4.022381E+05  3.609727E+05       41265.4       10.3443     0.0808834       1.09799      0.185006      0.243263    0.00536309    -0.0146693    0.00837298       1.04312       Opttag_D
      72       81.6143      0.962950       1.00000  0.000000E+00       1.00000  0.000000E+00      0.941367       100.508       1.00000       35.3358  0.000000E+00      -1.24523  0.000000E+00      -1.24523  0.000000E+00  0.000000E+00       2.49046       82.3604    -0.0687431        1       Scatag_D
      72       1976.89       5942.01  0.000000E+00  0.000000E+00       2693.75  0.000000E+00       2388.79       8454.11  0.000000E+00       13095.1       50.6921       6664.06 -1.000000E+10 -1.000000E+10 -1.000000E+10 -1.000000E+10 -1.000000E+10  0.000000E+00  0.000000E+00  0.000000E+00  0.000000E+00  0.000000E+00  0.000000E+00  0.000000E+00  0.000000E+00  0.000000E+00  0.000000E+00  0.000000E+00  0.000000E+00       Funtag_D
      73  4.022192E+05  3.609440E+05       41275.2       17.8223     0.0833266       1.12604      0.184977      0.243250    0.00544176    -0.0147130    0.00837489       1.04384       Opttag_D
      73       81.6474      0.962985       1.00000  0.000000E+00       1.00000  0.000000E+00      0.941401       100.455       1.00000       35.3247  0.000000E+00      -1.24860  0.000000E+00      -1.24860  0.000000E+00  0.000000E+00       2.49720       82.3604    -0.0687431        1       Scatag_D
      73       1977.79       5950.16  0.000000E+00  0.000000E+00       2695.93  0.000000E+00       2387.04       8454.72  0.000000E+00       13099.3       50.6920       6659.57 -1.000000E+10 -1.000000E+10 -1.000000E+10 -1.000000E+10 -1.000000E+10  0.000000E+00  0.000000E+00  0.000000E+00  0.000000E+00  0.000000E+00  0.000000E+00  0.000000E+00  0.000000E+00  0.000000E+00  0.000000E+00  0.000000E+00  0.000000E+00       Funtag_D
      74  4.021896E+05  3.609397E+05       41249.9       9.97500     0.0836263       1.12963      0.184974      0.243251    0.00545375    -0.0147346    0.00837415       1.04383       Opttag_D
      74       81.6513      0.962925       1.00000  0.000000E+00       1.00000  0.000000E+00      0.941405       100.447       1.00000       35.3246  0.000000E+00      -1.24890  0.000000E+00      -1.24890  0.000000E+00  0.000000E+00       2.49779       82.3604    -0.0687431        1       Scatag_D
      74       1977.44       5950.04  0.000000E+00  0.000000E+00       2696.20  0.000000E+00       2386.86       8429.79  0.000000E+00       13099.8       50.7420       6658.98 -1.000000E+10 -1.000000E+10 -1.000000E+10 -1.000000E+10 -1.000000E+10  0.000000E+00  0.000000E+00  0.000000E+00  0.000000E+00  0.000000E+00  0.000000E+00  0.000000E+00  0.000000E+00  0.000000E+00  0.000000E+00  0.000000E+00  0.000000E+00       Funtag_D
      75  4.021709E+05  3.609315E+05       41239.3       7.87636     0.0844188       1.13874      0.184968      0.243255    0.00547611    -0.0147821    0.00837558       1.04405       Opttag_D
      75       81.6618      0.962873       1.00000  0.000000E+00       1.00000  0.000000E+00      0.941416       100.426       1.00000       35.3238  0.000000E+00      -1.24986  0.000000E+00      -1.24986  0.000000E+00  0.000000E+00       2.49973       82.3604    -0.0687431        1       Scatag_D
      75       1978.12       5952.57  0.000000E+00  0.000000E+00       2696.89  0.000000E+00       2386.50       8415.33  0.000000E+00       13101.4       50.8286       6657.74 -1.000000E+10 -1.000000E+10 -1.000000E+10 -1.000000E+10 -1.000000E+10  0.000000E+00  0.000000E+00  0.000000E+00  0.000000E+00  0.000000E+00  0.000000E+00  0.000000E+00  0.000000E+00  0.000000E+00  0.000000E+00  0.000000E+00  0.000000E+00       Funtag_D
      76  4.021521E+05  3.609185E+05       41233.6       7.78195     0.0857272       1.15248      0.184956      0.243256    0.00551168    -0.0148419    0.00837788       1.04442       Opttag_D
      76       81.6794      0.962826       1.00000  0.000000E+00       1.00000  0.000000E+00      0.941434       100.392       1.00000       35.3213  0.000000E+00      -1.25143  0.000000E+00      -1.25143  0.000000E+00  0.000000E+00       2.50286       82.3604    -0.0687431        1       Scatag_D
      76       1979.21       5956.73  0.000000E+00  0.000000E+00       2697.98  0.000000E+00       2385.96       8403.29  0.000000E+00       13104.0       50.9044       6655.47 -1.000000E+10 -1.000000E+10 -1.000000E+10 -1.000000E+10 -1.000000E+10  0.000000E+00  0.000000E+00  0.000000E+00  0.000000E+00  0.000000E+00  0.000000E+00  0.000000E+00  0.000000E+00  0.000000E+00  0.000000E+00  0.000000E+00  0.000000E+00       Funtag_D
      77  4.021393E+05  3.608912E+05       41248.1       17.4398     0.0885751       1.17954      0.184937      0.243271    0.00558679    -0.0149630    0.00838245       1.04544       Opttag_D
      77       81.7182      0.962696       1.00000  0.000000E+00       1.00000  0.000000E+00      0.941476       100.310       1.00000       35.3172  0.000000E+00      -1.25428  0.000000E+00      -1.25428  0.000000E+00  0.000000E+00       2.50857       82.3604    -0.0687431        1       Scatag_D
      77       1981.37       5968.46  0.000000E+00  0.000000E+00       2700.22  0.000000E+00       2385.41       8401.31  0.000000E+00       13110.4       50.9905       6649.96 -1.000000E+10 -1.000000E+10 -1.000000E+10 -1.000000E+10 -1.000000E+10  0.000000E+00  0.000000E+00  0.000000E+00  0.000000E+00  0.000000E+00  0.000000E+00  0.000000E+00  0.000000E+00  0.000000E+00  0.000000E+00  0.000000E+00  0.000000E+00       Funtag_D
      78  4.021085E+05  3.608863E+05       41222.2       7.20980     0.0892246       1.18514      0.184932      0.243276    0.00560021    -0.0149863    0.00838224       1.04543       Opttag_D
      78       81.7273      0.962700       1.00000  0.000000E+00       1.00000  0.000000E+00      0.941485       100.293       1.00000       35.3161  0.000000E+00      -1.25510  0.000000E+00      -1.25510  0.000000E+00  0.000000E+00       2.51020       82.3604    -0.0687431        1       Scatag_D
      78       1981.27       5968.30  0.000000E+00  0.000000E+00       2700.65  0.000000E+00       2385.36       8375.27  0.000000E+00       13111.8       50.9840       6648.52 -1.000000E+10 -1.000000E+10 -1.000000E+10 -1.000000E+10 -1.000000E+10  0.000000E+00  0.000000E+00  0.000000E+00  0.000000E+00  0.000000E+00  0.000000E+00  0.000000E+00  0.000000E+00  0.000000E+00  0.000000E+00  0.000000E+00  0.000000E+00       Funtag_D
      79  4.020949E+05  3.608813E+05       41213.6       6.12266     0.0895055       1.18649      0.184929      0.243279    0.00561393    -0.0149923    0.00838128       1.04547       Opttag_D
      79       81.7318      0.962627       1.00000  0.000000E+00       1.00000  0.000000E+00      0.941490       100.282       1.00000       35.3142  0.000000E+00      -1.25543  0.000000E+00      -1.25543  0.000000E+00  0.000000E+00       2.51086       82.3604    -0.0687431        1       Scatag_D
      79       1980.81       5968.80  0.000000E+00  0.000000E+00       2700.76  0.000000E+00       2385.48       8367.53  0.000000E+00       13112.1       50.9358       6647.17 -1.000000E+10 -1.000000E+10 -1.000000E+10 -1.000000E+10 -1.000000E+10  0.000000E+00  0.000000E+00  0.000000E+00  0.000000E+00  0.000000E+00  0.000000E+00  0.000000E+00  0.000000E+00  0.000000E+00  0.000000E+00  0.000000E+00  0.000000E+00       Funtag_D
      80  4.020761E+05  3.608683E+05       41207.8       7.64092     0.0904120       1.19272      0.184920      0.243295    0.00564950    -0.0150254    0.00838037       1.04571       Opttag_D
      80       81.7453      0.962467       1.00000  0.000000E+00       1.00000  0.000000E+00      0.941503       100.253       1.00000       35.3112  0.000000E+00      -1.25640  0.000000E+00      -1.25640  0.000000E+00  0.000000E+00       2.51279       82.3604    -0.0687431        1       Scatag_D
      80       1980.38       5971.55  0.000000E+00  0.000000E+00       2701.22  0.000000E+00       2385.79       8360.20  0.000000E+00       13113.7       50.8735       6644.07 -1.000000E+10 -1.000000E+10 -1.000000E+10 -1.000000E+10 -1.000000E+10  0.000000E+00  0.000000E+00  0.000000E+00  0.000000E+00  0.000000E+00  0.000000E+00  0.000000E+00  0.000000E+00  0.000000E+00  0.000000E+00  0.000000E+00  0.000000E+00       Funtag_D
      81  4.020493E+05  3.608436E+05       41205.7       13.1663     0.0920720       1.20444      0.184904      0.243333    0.00571749    -0.0151347    0.00837923       1.04618       Opttag_D
      81       81.7705      0.962160       1.00000  0.000000E+00       1.00000  0.000000E+00      0.941525       100.204       1.00000       35.3068  0.000000E+00      -1.25843  0.000000E+00      -1.25843  0.000000E+00  0.000000E+00       2.51686       82.3604    -0.0687431        1       Scatag_D
      81       1979.84       5976.85  0.000000E+00  0.000000E+00       2702.01  0.000000E+00       2386.57       8353.84  0.000000E+00       13116.6       50.8587       6639.09 -1.000000E+10 -1.000000E+10 -1.000000E+10 -1.000000E+10 -1.000000E+10  0.000000E+00  0.000000E+00  0.000000E+00  0.000000E+00  0.000000E+00  0.000000E+00  0.000000E+00  0.000000E+00  0.000000E+00  0.000000E+00  0.000000E+00  0.000000E+00       Funtag_D
      82  4.020182E+05  3.608176E+05       41200.6       10.5018     0.0943101       1.22025      0.184889      0.243391    0.00578884    -0.0152809    0.00837942       1.04677       Opttag_D
      82       81.8038      0.961919       1.00000  0.000000E+00       1.00000  0.000000E+00      0.941554       100.140       1.00000       35.3053  0.000000E+00      -1.26087  0.000000E+00      -1.26087  0.000000E+00  0.000000E+00       2.52173       82.3604    -0.0687431        1       Scatag_D
      82       1979.94       5983.63  0.000000E+00  0.000000E+00       2703.11  0.000000E+00       2387.89       8339.60  0.000000E+00       13121.8       50.8965       6633.81 -1.000000E+10 -1.000000E+10 -1.000000E+10 -1.000000E+10 -1.000000E+10  0.000000E+00  0.000000E+00  0.000000E+00  0.000000E+00  0.000000E+00  0.000000E+00  0.000000E+00  0.000000E+00  0.000000E+00  0.000000E+00  0.000000E+00  0.000000E+00       Funtag_D
      83  4.019962E+05  3.608038E+05       41192.3       7.94688     0.0952536       1.22595      0.184878      0.243425    0.00582655    -0.0153679    0.00837887       1.04700       Opttag_D
      83       81.8185      0.961769       1.00000  0.000000E+00       1.00000  0.000000E+00      0.941565       100.115       1.00000       35.3052  0.000000E+00      -1.26193  0.000000E+00      -1.26193  0.000000E+00  0.000000E+00       2.52387       82.3604    -0.0687431        1       Scatag_D
      83       1979.67       5986.21  0.000000E+00  0.000000E+00       2703.53  0.000000E+00       2388.62       8328.04  0.000000E+00       13123.5       50.9815       6631.74 -1.000000E+10 -1.000000E+10 -1.000000E+10 -1.000000E+10 -1.000000E+10  0.000000E+00  0.000000E+00  0.000000E+00  0.000000E+00  0.000000E+00  0.000000E+00  0.000000E+00  0.000000E+00  0.000000E+00  0.000000E+00  0.000000E+00  0.000000E+00       Funtag_D
      84  4.019718E+05  3.607813E+05       41190.6       8.75489     0.0967169       1.23309      0.184860      0.243487    0.00588844    -0.0155215    0.00837957       1.04733       Opttag_D
      84       81.8421      0.961528       1.00000  0.000000E+00       1.00000  0.000000E+00      0.941580       100.076       1.00000       35.3054  0.000000E+00      -1.26360  0.000000E+00      -1.26360  0.000000E+00  0.000000E+00       2.52720       82.3604    -0.0687431        1       Scatag_D
      84       1980.00       5990.02  0.000000E+00  0.000000E+00       2704.14  0.000000E+00       2389.86       8320.52  0.000000E+00       13126.1       51.1594       6628.77 -1.000000E+10 -1.000000E+10 -1.000000E+10 -1.000000E+10 -1.000000E+10  0.000000E+00  0.000000E+00  0.000000E+00  0.000000E+00  0.000000E+00  0.000000E+00  0.000000E+00  0.000000E+00  0.000000E+00  0.000000E+00  0.000000E+00  0.000000E+00       Funtag_D
      85  4.019568E+05  3.607641E+05       41192.7       9.62579     0.0978515       1.23746      0.184847      0.243528    0.00593550    -0.0156193    0.00838055       1.04754       Opttag_D
      85       81.8610      0.961376       1.00000  0.000000E+00       1.00000  0.000000E+00      0.941591       100.047       1.00000       35.3060  0.000000E+00      -1.26471  0.000000E+00      -1.26471  0.000000E+00  0.000000E+00       2.52943       82.3604    -0.0687431        1       Scatag_D
      85       1980.47       5992.41  0.000000E+00  0.000000E+00       2704.58  0.000000E+00       2390.88       8318.58  0.000000E+00       13128.0       51.2707       6626.48 -1.000000E+10 -1.000000E+10 -1.000000E+10 -1.000000E+10 -1.000000E+10  0.000000E+00  0.000000E+00  0.000000E+00  0.000000E+00  0.000000E+00  0.000000E+00  0.000000E+00  0.000000E+00  0.000000E+00  0.000000E+00  0.000000E+00  0.000000E+00       Funtag_D
      86  4.019416E+05  3.607589E+05       41182.8       7.75537     0.0979664       1.23648      0.184843      0.243532    0.00594993    -0.0156170    0.00837972       1.04747       Opttag_D
      86       81.8639      0.961311       1.00000  0.000000E+00       1.00000  0.000000E+00      0.941592       100.044       1.00000       35.3050  0.000000E+00      -1.26473  0.000000E+00      -1.26473  0.000000E+00  0.000000E+00       2.52945       82.3604    -0.0687431        1       Scatag_D
      86       1980.07       5991.58  0.000000E+00  0.000000E+00       2704.61  0.000000E+00       2390.99       8310.72  0.000000E+00       13127.7       51.2579       6625.86 -1.000000E+10 -1.000000E+10 -1.000000E+10 -1.000000E+10 -1.000000E+10  0.000000E+00  0.000000E+00  0.000000E+00  0.000000E+00  0.000000E+00  0.000000E+00  0.000000E+00  0.000000E+00  0.000000E+00  0.000000E+00  0.000000E+00  0.000000E+00       Funtag_D
      87  4.019070E+05  3.607328E+05       41174.2       8.69372     0.0992771       1.23785      0.184821      0.243574    0.00602138    -0.0156436    0.00838006       1.04750       Opttag_D
      87       81.8885      0.961093       1.00000  0.000000E+00       1.00000  0.000000E+00      0.941603       100.011       1.00000       35.3038  0.000000E+00      -1.26549  0.000000E+00      -1.26549  0.000000E+00  0.000000E+00       2.53097       82.3604    -0.0687431        1       Scatag_D
      87       1980.24       5991.92  0.000000E+00  0.000000E+00       2705.10  0.000000E+00       2392.27       8302.29  0.000000E+00       13129.1       51.2139       6622.08 -1.000000E+10 -1.000000E+10 -1.000000E+10 -1.000000E+10 -1.000000E+10  0.000000E+00  0.000000E+00  0.000000E+00  0.000000E+00  0.000000E+00  0.000000E+00  0.000000E+00  0.000000E+00  0.000000E+00  0.000000E+00  0.000000E+00  0.000000E+00       Funtag_D
      88  4.018925E+05  3.607085E+05       41184.0       13.8686      0.100812       1.24176      0.184801      0.243616    0.00608816    -0.0157116    0.00838114       1.04764       Opttag_D
      88       81.9161      0.960954       1.00000  0.000000E+00       1.00000  0.000000E+00      0.941615       99.9775       1.00000       35.3048  0.000000E+00      -1.26664  0.000000E+00      -1.26664  0.000000E+00  0.000000E+00       2.53329       82.3604    -0.0687431        1       Scatag_D
      88       1980.75       5993.55  0.000000E+00  0.000000E+00       2705.72  0.000000E+00       2393.99       8308.96  0.000000E+00       13131.7       51.1987       6618.19 -1.000000E+10 -1.000000E+10 -1.000000E+10 -1.000000E+10 -1.000000E+10  0.000000E+00  0.000000E+00  0.000000E+00  0.000000E+00  0.000000E+00  0.000000E+00  0.000000E+00  0.000000E+00  0.000000E+00  0.000000E+00  0.000000E+00  0.000000E+00       Funtag_D
      89  4.018721E+05  3.606990E+05       41173.1       9.28950      0.101476       1.24376      0.184794      0.243635    0.00611428    -0.0157291    0.00838114       1.04770       Opttag_D
      89       81.9280      0.960917       1.00000  0.000000E+00       1.00000  0.000000E+00      0.941620       99.9636       1.00000       35.3059  0.000000E+00      -1.26724  0.000000E+00      -1.26724  0.000000E+00  0.000000E+00       2.53449       82.3604    -0.0687431        1       Scatag_D
      89       1980.75       5994.27  0.000000E+00  0.000000E+00       2705.98  0.000000E+00       2394.74       8296.91  0.000000E+00       13132.9       51.1776       6616.36 -1.000000E+10 -1.000000E+10 -1.000000E+10 -1.000000E+10 -1.000000E+10  0.000000E+00  0.000000E+00  0.000000E+00  0.000000E+00  0.000000E+00  0.000000E+00  0.000000E+00  0.000000E+00  0.000000E+00  0.000000E+00  0.000000E+00  0.000000E+00       Funtag_D
      90  4.018423E+05  3.606748E+05       41167.6       6.72865      0.103312       1.24940      0.184774      0.243692    0.00618075    -0.0157909    0.00838222       1.04795       Opttag_D
      90       81.9609      0.960865       1.00000  0.000000E+00       1.00000  0.000000E+00      0.941630       99.9286       1.00000       35.3104  0.000000E+00      -1.26908  0.000000E+00      -1.26908  0.000000E+00  0.000000E+00       2.53816       82.3604    -0.0687431        1       Scatag_D
      90       1981.26       5997.14  0.000000E+00  0.000000E+00       2706.71  0.000000E+00       2396.87       8286.78  0.000000E+00       13136.4       51.1534       6611.28 -1.000000E+10 -1.000000E+10 -1.000000E+10 -1.000000E+10 -1.000000E+10  0.000000E+00  0.000000E+00  0.000000E+00  0.000000E+00  0.000000E+00  0.000000E+00  0.000000E+00  0.000000E+00  0.000000E+00  0.000000E+00  0.000000E+00  0.000000E+00       Funtag_D
      91  4.018258E+05  3.606582E+05       41167.6       6.82413      0.104473       1.25164      0.184758      0.243723    0.00622618    -0.0158324    0.00838265       1.04814       Opttag_D
      91       81.9826      0.960834       1.00000  0.000000E+00       1.00000  0.000000E+00      0.941635       99.9079       1.00000       35.3141  0.000000E+00      -1.27046  0.000000E+00      -1.27046  0.000000E+00  0.000000E+00       2.54092       82.3604    -0.0687431        1       Scatag_D
      91       1981.46       5999.25  0.000000E+00  0.000000E+00       2707.18  0.000000E+00       2398.25       8284.35  0.000000E+00       13138.1       51.1798       6607.76 -1.000000E+10 -1.000000E+10 -1.000000E+10 -1.000000E+10 -1.000000E+10  0.000000E+00  0.000000E+00  0.000000E+00  0.000000E+00  0.000000E+00  0.000000E+00  0.000000E+00  0.000000E+00  0.000000E+00  0.000000E+00  0.000000E+00  0.000000E+00       Funtag_D
      92  4.018087E+05  3.606343E+05       41174.3       11.1680      0.106170       1.25355      0.184737      0.243755    0.00629173    -0.0158689    0.00838300       1.04845       Opttag_D
      92       82.0156      0.960829       1.00000  0.000000E+00       1.00000  0.000000E+00      0.941638       99.8799       1.00000       35.3210  0.000000E+00      -1.27264  0.000000E+00      -1.27264  0.000000E+00  0.000000E+00       2.54527       82.3604    -0.0687431        2       Scatag_D
      92       1981.63       6002.83  0.000000E+00  0.000000E+00       2707.88  0.000000E+00       2400.22       8287.81  0.000000E+00       13140.4       51.2298       6602.29 -1.000000E+10 -1.000000E+10 -1.000000E+10 -1.000000E+10 -1.000000E+10  0.000000E+00  0.000000E+00  0.000000E+00  0.000000E+00  0.000000E+00  0.000000E+00  0.000000E+00  0.000000E+00  0.000000E+00  0.000000E+00  0.000000E+00  0.000000E+00       Funtag_D
      93  4.017841E+05  3.606122E+05       41171.9       6.82025      0.107611       1.25743      0.184718      0.243775    0.00635251    -0.0159061    0.00838367       1.04868       Opttag_D
      93       82.0445      0.960806       1.00000  0.000000E+00       1.00000  0.000000E+00      0.941639       99.8572       1.00000       35.3278  0.000000E+00      -1.27466  0.000000E+00      -1.27466  0.000000E+00  0.000000E+00       2.54933       82.3604    -0.0687431        1       Scatag_D
      93       1981.94       6005.52  0.000000E+00  0.000000E+00       2708.54  0.000000E+00       2401.83       8283.19  0.000000E+00       13142.2       51.3026       6597.32 -1.000000E+10 -1.000000E+10 -1.000000E+10 -1.000000E+10 -1.000000E+10  0.000000E+00  0.000000E+00  0.000000E+00  0.000000E+00  0.000000E+00  0.000000E+00  0.000000E+00  0.000000E+00  0.000000E+00  0.000000E+00  0.000000E+00  0.000000E+00       Funtag_D
      94  4.017666E+05  3.605963E+05       41170.3       6.79608      0.108247       1.26292      0.184705      0.243767    0.00639611    -0.0158955    0.00838370       1.04879       Opttag_D
      94       82.0594      0.960704       1.00000  0.000000E+00       1.00000  0.000000E+00      0.941636       99.8485       1.00000       35.3309  0.000000E+00      -1.27588  0.000000E+00      -1.27588  0.000000E+00  0.000000E+00       2.55176       82.3604    -0.0687431        1       Scatag_D
      94       1981.96       6006.75  0.000000E+00  0.000000E+00       2708.82  0.000000E+00       2402.18       8282.89  0.000000E+00       13142.3       51.3397       6594.04 -1.000000E+10 -1.000000E+10 -1.000000E+10 -1.000000E+10 -1.000000E+10  0.000000E+00  0.000000E+00  0.000000E+00  0.000000E+00  0.000000E+00  0.000000E+00  0.000000E+00  0.000000E+00  0.000000E+00  0.000000E+00  0.000000E+00  0.000000E+00       Funtag_D
      95  4.017389E+05  3.605622E+05       41176.6       10.4705      0.109585       1.27349      0.184675      0.243741    0.00648972    -0.0158487    0.00838663       1.04904       Opttag_D
      95       82.0916      0.960492       1.00000  0.000000E+00       1.00000  0.000000E+00      0.941627       99.8318       1.00000       35.3378  0.000000E+00      -1.27844  0.000000E+00      -1.27844  0.000000E+00  0.000000E+00       2.55687       82.3604    -0.0687431        1       Scatag_D
      95       1983.34       6009.57  0.000000E+00  0.000000E+00       2709.48  0.000000E+00       2402.58       8289.83  0.000000E+00       13143.0       51.3716       6587.46 -1.000000E+10 -1.000000E+10 -1.000000E+10 -1.000000E+10 -1.000000E+10  0.000000E+00  0.000000E+00  0.000000E+00  0.000000E+00  0.000000E+00  0.000000E+00  0.000000E+00  0.000000E+00  0.000000E+00  0.000000E+00  0.000000E+00  0.000000E+00       Funtag_D
      96  4.017148E+05  3.605256E+05       41189.2       12.6598      0.110918       1.28266      0.184644      0.243702    0.00659003    -0.0157776    0.00839144       1.04930       Opttag_D
      96       82.1250      0.960244       1.00000  0.000000E+00       1.00000  0.000000E+00      0.941618       99.8161       1.00000       35.3456  0.000000E+00      -1.28090  0.000000E+00      -1.28090  0.000000E+00  0.000000E+00       2.56180       82.3604    -0.0687431        1       Scatag_D
      96       1985.62       6012.61  0.000000E+00  0.000000E+00       2710.24  0.000000E+00       2402.64       8299.58  0.000000E+00       13145.1       51.3631       6582.05 -1.000000E+10 -1.000000E+10 -1.000000E+10 -1.000000E+10 -1.000000E+10  0.000000E+00  0.000000E+00  0.000000E+00  0.000000E+00  0.000000E+00  0.000000E+00  0.000000E+00  0.000000E+00  0.000000E+00  0.000000E+00  0.000000E+00  0.000000E+00       Funtag_D
      97  4.016912E+05  3.605158E+05       41175.4       9.27644      0.110959       1.28126      0.184637      0.243675    0.00661696    -0.0157319    0.00839238       1.04922       Opttag_D
      97       82.1291      0.960094       1.00000  0.000000E+00       1.00000  0.000000E+00      0.941615       99.8172       1.00000       35.3450  0.000000E+00      -1.28142  0.000000E+00      -1.28142  0.000000E+00  0.000000E+00       2.56283       82.3604    -0.0687431        1       Scatag_D
      97       1986.06       6011.72  0.000000E+00  0.000000E+00       2710.26  0.000000E+00       2401.76       8287.90  0.000000E+00       13145.5       51.3172       6580.91 -1.000000E+10 -1.000000E+10 -1.000000E+10 -1.000000E+10 -1.000000E+10  0.000000E+00  0.000000E+00  0.000000E+00  0.000000E+00  0.000000E+00  0.000000E+00  0.000000E+00  0.000000E+00  0.000000E+00  0.000000E+00  0.000000E+00  0.000000E+00       Funtag_D
      98  4.016610E+05  3.604926E+05       41168.4       8.11184      0.111358       1.28105      0.184619      0.243624    0.00668079    -0.0156476    0.00839715       1.04918       Opttag_D
      98       82.1439      0.959837       1.00000  0.000000E+00       1.00000  0.000000E+00      0.941611       99.8146       1.00000       35.3453  0.000000E+00      -1.28299  0.000000E+00      -1.28299  0.000000E+00  0.000000E+00       2.56597       82.3604    -0.0687431        1       Scatag_D
      98       1988.32       6011.26  0.000000E+00  0.000000E+00       2710.52  0.000000E+00       2400.15       8280.39  0.000000E+00       13148.0       51.2547       6578.56 -1.000000E+10 -1.000000E+10 -1.000000E+10 -1.000000E+10 -1.000000E+10  0.000000E+00  0.000000E+00  0.000000E+00  0.000000E+00  0.000000E+00  0.000000E+00  0.000000E+00  0.000000E+00  0.000000E+00  0.000000E+00  0.000000E+00  0.000000E+00       Funtag_D
      99  4.016445E+05  3.604729E+05       41171.6       9.02584      0.111857       1.28232      0.184599      0.243585    0.00673486    -0.0155843    0.00840038       1.04926       Opttag_D
      99       82.1595      0.959685       1.00000  0.000000E+00       1.00000  0.000000E+00      0.941609       99.8097       1.00000       35.3470  0.000000E+00      -1.28477  0.000000E+00      -1.28477  0.000000E+00  0.000000E+00       2.56953       82.3604    -0.0687431        1       Scatag_D
      99       1989.85       6012.12  0.000000E+00  0.000000E+00       2710.84  0.000000E+00       2399.23       8280.77  0.000000E+00       13150.5       51.2523       6577.03 -1.000000E+10 -1.000000E+10 -1.000000E+10 -1.000000E+10 -1.000000E+10  0.000000E+00  0.000000E+00  0.000000E+00  0.000000E+00  0.000000E+00  0.000000E+00  0.000000E+00  0.000000E+00  0.000000E+00  0.000000E+00  0.000000E+00  0.000000E+00       Funtag_D

   MaxLik Scaling Cycle 0106

   Wilson plot

   Statistics in resolution bins after refinement cycle 0003
```


|  |  |
| --- | --- |
|  | - Rxpct factors plot- Rxpct factors table- <Log-Lik Gain>- Ln Sigma A- Correlation Coefficients |


```
   Iters     Tot_Funct    Xray_Funct    Geom_Funct          Grms     rmsD2init     maxD2init             R         Rfree           LLG       LLGfree     rms(bond)    rms(angle)
   Iters             K   B_IMPF_FRAG        K_MISS   B_TEMP_MISS   K_IMPF_MISS   B_IMPF_MISS        K_SOLV   B_TEMP_SOLV   K_IMPF_SOLV   B_IMPF_SOLV        B_TEMP     B_TEMP_11     B_TEMP_12     B_TEMP_22     B_TEMP_13     B_TEMP_23     B_TEMP_33      K_WILSON      B_WILSON      FGCYCLES
   Iters          BOND         ANGLE      IMPROPER       TORSION    SINTORSION      TRIGONAL        PLANAR  BCORRELATION       CONTACT     IDEALDIST        CHIRAL    SIMILARITY       OCCSSUM  UTILDISTANCE     UTILANGLE   UTILTORSION        RHOFIT        ADP-01        ADP-02        ADP-03        ADP-04        ADP-05        ADP-06        ADP-07        ADP-08        ADP-09        ADP-10      RHOFITPC            QM
     100  4.016252E+05  3.604524E+05       41172.8       7.29214      0.112494       1.28466      0.184579      0.243548    0.00679107    -0.0155229    0.00840317       1.04933       Opttag_D
     100       82.1780      0.959578       1.00000  0.000000E+00       1.00000  0.000000E+00      0.941610       99.8022       1.00000       35.3496  0.000000E+00      -1.28695  0.000000E+00      -1.28695  0.000000E+00  0.000000E+00       2.57390       82.3604    -0.0687431        1       Scatag_D
     100       1991.17       6012.95  0.000000E+00  0.000000E+00       2711.24  0.000000E+00       2398.50       8278.22  0.000000E+00       13153.6       51.2696       6575.82 -1.000000E+10 -1.000000E+10 -1.000000E+10 -1.000000E+10 -1.000000E+10  0.000000E+00  0.000000E+00  0.000000E+00  0.000000E+00  0.000000E+00  0.000000E+00  0.000000E+00  0.000000E+00  0.000000E+00  0.000000E+00  0.000000E+00  0.000000E+00       Funtag_D
   Geometry Restraint Deviations SCREEN Information

 
 No -bcorrel_zeroocchydrogens_factor flag found - keeping default: will set B for OCC=0 hydrogens to    1.200 times heavy-atom B


 TLS information. Total number of groups:     2


 Group   Natoms    FixRB   FixTLS   Group Tag    Group Spec
     1     2783      Y        Y     tls1    { A|* }
     2     2770      Y        Y     tls2    { B|* }
 
NOTE BUSTER_TLS_SET tls1 { A|* }
NOTE BUSTER_TLS_T   tls1     -2.44720E-01 -1.33885E-01  3.49464E-01 -7.26320E-02  1.36549E-02 -3.31073E-02
NOTE BUSTER_TLS_L   tls1      2.21033E+00  2.43686E+00  1.90206E+00 -4.70482E-01 -1.42181E+00  6.26775E-01
NOTE BUSTER_TLS_S   tls1      1.26526E-01 -3.75851E-01  4.70780E-01 -3.29027E-01  1.72095E-01 -3.71904E-01  6.80978E-02 -3.47299E-01
NOTE BUSTER_TLS_O   tls1     -2.31731E+01 -8.40215E+01  1.10770E+01
NOTE BUSTER_TLS_FIX tls1 ALL
 
NOTE BUSTER_TLS_SET tls2 { B|* }
NOTE BUSTER_TLS_T   tls2     -3.51018E-01 -2.65873E-01  4.05020E-01  2.48330E-02  3.11379E-02 -4.04722E-02
NOTE BUSTER_TLS_L   tls2      1.80307E+00  1.69393E+00  1.39140E+00  8.83117E-01 -1.11732E+00 -9.56109E-01
NOTE BUSTER_TLS_S   tls2     -1.42162E-02  9.70000E-02 -1.62700E-01  8.39808E-02  2.10687E-01  5.67053E-02 -9.55320E-02  7.37692E-02
NOTE BUSTER_TLS_O   tls2      2.22040E+01 -7.22942E+01  3.58164E+00
NOTE BUSTER_TLS_FIX tls2 ALL

 For final position, Geometry function summary
     term                           number     weight       rms        GooF      function     fn/numb
     ================================================================================================
     BOND bond lengths (angs)       5668       2.000      0.0084       0.419      1991.17       0.351
     ANGL bond angles (degs)        7691       2.000        1.05       0.625      6012.95       0.782
     TORS split-harmonic tors       1275       0.000       19.09       1.200         0.00       0.000
     SINTOR sinusoidal tors         1275       2.000       19.09                  2711.24       2.126
     PLAN general plane (angs)       967       5.000      0.0141       0.704      2398.50       2.480
     BCORrelations (angs^2)         5668      10.000       1.071       0.382      8278.22       1.461
     CONT (bad contacts)               0       5.000                                 0.00
     IDEAL(ideal-dist contact)      5666       4.000                             13153.61
     CHIRAL (gelly semiharmon)       740       5.000                                51.27
     SIM similarity restraint for NCS and/or TARGET                               6575.82
     OCCSUM restraint on sum of occupanies                                           0.00       as none defined
     DISTAN utility restraints interatomic distance                                  0.00       as none defined
     UTILANGLE utility angle restraints                                              0.00       as none defined
     UTILTOR utility restraints on torsion angles                                    0.00       as none defined
     Peptide omega torsion angles                           2.88 degs - monitor only

 Weighted rms      0.0084         1.049  (temporary rms bond and angle for BUSTER to pick up!)
 Weighted intra-residual rms(bond) for StdProtein (excluding H)      0.0082
 Weighted intra-residual rms(bond) for Buffer     (excluding H)      0.0030
 Outputing geometry progress graph in plotmtv format to file:Geom_Res.mtv

 ############### Running final geometry/screen analysis (screen_final.txt)

 s/r gelly normal termination
```

Statistics and scale factors vs. cycle number
```

|  |  |  |
| --- | --- | --- |
|  | - Rxpct factors- <Log-Lik Gain>- GEOM Residuals- Ln Sigma_A_ | - Scale Factors- B Factors- Imperfection Factors |

```
   Final Geometry Information


---


### 2. Final results

Statistics and scale factors vs. cycle number
```

|  |  |  |
| --- | --- | --- |
|  | - Rxpct factors- <Log-Lik Gain>- GEOM Residuals- Ln Sigma_A_ | - Scale Factors- B Factors- Imperfection Factors |

```
   Average Figure of Merit
   Structure Factor Amplitudes


 # normal termination

  Program stopped.
```

closesok message: time spent in tree = 1823.81 (user) 27.75 (sys)

---

# Normal termination

```
 Resources usage summary:
     Time spent in client ...................  1823.81 (user)       27.77 (system)
     Time spent in scripts (    27 calls) ...     2.21 (user)        0.17 (system)
     Time spent in server ...................     0.00 (user)        0.00 (system)

     Max resident memory used (kb) .......... 378212
     Number of page faults .................. 0 (major)    374550 (minor)
     Number of context switches ............. 2105306 (niced)    8078 (other)
```
